# Children’s Comprehension of Sentences with Focus Particles and the Role of Cognitive Control: An Eye Tracking Study with German-Learning 4-Year-Olds

**DOI:** 10.1371/journal.pone.0149870

**Published:** 2016-03-01

**Authors:** Barbara Höhle, Tom Fritzsche, Anja Müller

**Affiliations:** 1 Department of Linguistics, Research Center Cognitive Science, Universität Potsdam, Potsdam, Germany; 2 Institute for Psycholinguistics, Goethe-Universität Frankfurt, Frankfurt, Germany; University of Leicester, UNITED KINGDOM

## Abstract

Children’s interpretations of sentences containing focus particles do not seem adult-like until school age. This study investigates how German 4-year-old children comprehend sentences with the focus particle ‘nur’ (only) by using different tasks and controlling for the impact of general cognitive abilities on performance measures. Two sentence types with ‘only’ in either pre-subject or pre-object position were presented. Eye gaze data and verbal responses were collected via the visual world paradigm combined with a sentence-picture verification task. While the eye tracking data revealed an adult-like pattern of focus particle processing, the sentence-picture verification replicated previous findings of poor comprehension, especially for ‘only’ in pre-subject position. A second study focused on the impact of general cognitive abilities on the outcomes of the verification task. Working memory was related to children’s performance in both sentence types whereas inhibitory control was selectively related to the number of errors for sentences with ‘only’ in pre-subject position. These results suggest that children at the age of 4 years have the linguistic competence to correctly interpret sentences with focus particles, which–depending on specific task demands–may be masked by immature general cognitive abilities.

## Introduction

Children’s ability to comprehend sentences that contain focus particles (FPs) like *also*, *only*, or *even* has attracted increasing scientific attention in recent years. Numerous studies across languages as diverse as English, Japanese, and Mandarin Chinese have demonstrated that children up to school age still struggle with tasks intended to test their understanding of sentences containing these particles. What may make the understanding of these sentences challenging is the fact that knowledge from several linguistic domains must be available for their correct interpretation. To illustrate this, consider a sentence like in (1).

(1) *The zookeeper only gives the bananas to the bear.*

Syntactic properties of the sentence are relevant to restricting the scope domain of the FP, which structurally needs to c-command its potential associates [1, 2]. Thus, the FP in (1) can relate to the direct object *bananas*, creating the meaning that the zookeeper does not give anything else to the bear other than the bananas. It can also be related to the indirect object *bear*, creating the meaning that the zookeeper does not give bananas to anybody else other than the bear. Finally, it can associate to the whole VP, with the meaning that the zookeeper does not do anything else besides give bananas to the bear. To resolve this syntactic ambiguity, sentence focus must be considered, as FPs typically relate to the focused constituent of the sentence. In English, prosodic prominence is a major means of focus marking, thus the ambiguity created by the scope ambiguity can partially be resolved by relying on the accent pattern: an accent on the direct object rules out the meaning which results from the association of the FP to the indirect object whereas an accent on the indirect object excludes the meaning created by the association of the FP to the direct object.

Following Rooth [3, 4] we consider focus to indicate the presence of alternatives for the focused constituent in the (discourse) context that are relevant for the interpretation of a linguistic expression. Furthermore, the FP has a lexical meaning with *only* indicating that the statement about the focused constituent is restricted to this element and not true for the set of alternatives. Given the necessity to integrate information from different linguistic levels like syntax, prosody, pragmatics, and the lexicon, the causes of children’s problems in understanding these sentences could be multifaceted. Accordingly, present accounts to explain this developmental challenge are diverse: they propose either an insufficient integration of prosodic information into the parsing process [5–7], problems in identifying the scope domain of the focus particle due to the still developing syntactic system [8–10], or a still not adult-like pragmatic knowledge that may hinder children’s mental representation of alternatives [11].

In this paper we compare German-learning 4-years-olds’ comprehension of sentences with pre-subject vs. pre-object *only* in an eye tracking visual world paradigm and a picture-sentence verification task in order to shed more light on children’s processing of these sentences and thus contribute to the ongoing discussion about the cause underlying children’s non-adult-like performance with these sentences (Study 1). In addition to previous research, we include measures for cognitive control abilities (Study 2), which we consider to have a substantial impact on the processes involved in typical tasks assessing sentence comprehension [12]. Our study focuses on sentences with pre-subject or pre-object *only*, so the following literature review will be restricted to studies and findings on these kind of sentences.

Paterson et al. [11] tested groups of English-speaking children and adults on their interpretation of sentences such as *Only the fireman is holding a hose* and *The fireman is only holding a hose* as well as their counterpart *The fireman is holding a hose* without the particle. Specifically, they compared the performance with sentences including the FP to the corresponding sentences without any FP. In their task, the participants had to decide which pictures from a set of six alternatives matched a given sentence. Each set of pictures depicted the two same characters holding or not holding one or two different objects. They were set up in a way that the specific pattern of responses to all six members of a picture set would reveal whether the hearer had correctly considered the different scope restrictions for sentences with pre-subject or pre-object *only* and whether the FP had entered the sentence interpretation at all. For the sake of simplicity, we use the term ʻpre-objectʼ for all cases in which the FP does not occur before the subject although the object associated *only* can appear in different sentence positions in English: before the finite verb as in example (1) or after the finite verb as in the materials used for example in the studies by Paterson et al. [11] and Crain et al. [13]. For the youngest age groups (4- to 5-year-olds and 6- to 7-year-olds) Paterson and colleagues found that their major response pattern for all sentence types consisted in accepting the picture set that matched the reading of the sentence lacking the FP without any indications of differences between sentences with pre-subject and pre-object *only*. Based on these results they argue that children–due to their less developed pragmatic competence–may fail to mentally represent sets of alternatives, especially in conditions in which this set is not available from prior discourse context but has to be inferred from other sources. This account implies that interpreting a sentence with *only* depends on the degree to which the set of alternatives is salient within the given context–a factor that is not only assumed to affect children’s construction of the set of alternatives, but also adults’. Indeed, a later study by Paterson, Liversedge, White, Filik, & Jaz [14] with a reduced set of pictures revealed a lower amount of errors that indicate a failure to represent alternatives. Furthermore, in various studies carried out in different languages such as German [15–17], Dutch [18], and English [6, 19], children’s interpretation of sentences containing *only* improved when a verbal context provided an explicit introduction of the distinct sets of alternatives. These results support Paterson et al.’s [11] proposal that the availability of contextually given alternatives is important, especially for children’s correct interpretation of sentences with *only*.

But findings revealing an uneven performance in children’s interpretation of sentences in which the focus particle is related either to the subject or to the object of a sentence show that this approach does not fully cover the performance patterns that children exhibit with these sentences. While Paterson et al. [11] failed to find considerable differences between children’s performance on pre-subject and pre-object *only* sentences, other studies have reported an asymmetric pattern with better scores for pre-object compared to pre-subject *only* sentences across a number of different languages: English [9, 13, 19, 20], Mandarin Chinese [9, 10], Japanese [21], German [17], and European Portuguese [7]. One of the earliest findings is reported by Crain and colleagues [13]. They conducted a sentence-picture verification task with 3- to 6-year-old English-speaking children, who had to decide whether or not a sentence matched a picture. Overall, the children performed better in sentences containing pre-object *only* as in *The cat is only holding a flag* than in sentences containing pre-subject *only*. For the latter type of sentence they often accepted a picture that matched the meaning of a sentence with pre-object *only*.

More recent studies using the truth value judgment task (TVJT) [22], in which children have to judge whether a test sentence matches a story that had been acted out before, have replicated these findings with Mandarin-learning 4-year-old children [9, 10]. In this task, the children incorrectly rejected contextually appropriate pre-subject *only* sentences in 90% of the cases when the scenario involved a situation that falsified a pre-object *only* sentence (e.g. scenario: Mr. Pig gets a gold coin and a silver coin; Mr. Horse gets a gold coin; test sentence: *Only Mr*. *Pig got a silver coin*). Asked for the reason for their rejection, children often pointed out that–for the example above–Mr. Pig also got a gold coin. These justifications show that they had parsed the meaning contribution of the focus particle but in fact did not associate it to the sentence subject but to the object (or the VP).

Notley and colleagues [9] as well as Zhou and Crain [10] argue in favor of a syntactic explanation for children’s non-adult-like performance with pre-subject *only* sentences. They assume that children misanalyze SVO sentences with pre-subject *only* as if the particle took scope over the VP. More specifically, they claim that–unlike the adult grammar–children’s grammar allows pre-subject *only* to be analyzed as a sentential adverb which c-commands not only the subject but all the rest of the sentence, too. Under this account children’s non-adult-like interpretation of sentences with pre-subject *only* originates from their non-adult-like grammar. Evidence for this is provided by another study by Zhou and Crain [10]. They tested Mandarin-speaking children’s understanding of pre-subject *only* sentences with negation in the preverbal position. Based on Relativized Minimality [23, 24], Zhou and Crain argue that the negation particle (being of the same structural type as the focus particle analyzed as a sentential adverb) blocks the association between the FP and the VP and therefore enhanced performance is expected with these sentences (e.g. *Only the white dog didn’t climb up the big tree*, presented after a scenario with three dogs all having climbed up a small tree but only the black dog having been successful in climbing up the big tree). In this experiment the 4-year-old children showed the same correct rejection rates as adults for the pre-subject *only* sentences with negation (for which a pre-object *only* interpretation would not have been true in the given sentence) and justified their responses in the correct way (saying that there was another character in the story that did not perform the action). However, their performance in the sentences without negation was still significantly below that of the adult participants.

Müller et al. [17] tested German-learning 4- and 6-year-olds with a sentence-picture verification task on their understanding of pre-subject and pre-object *only* sentences and–as a control–on sentences without any FP. The analysis of the data focused on correct rejections of pictures that did not match the sentence, as only these responses were suited to uncovering the correct integration of the FP into sentence interpretation. Both age groups showed a significantly lower performance for the sentences containing the pre-subject *only* than for those with pre-object *only* even though the 6-year-olds outperformed the 4-year-olds. In addition to canonical SVO sentences, Müller, Höhle, and Schulz [25] also tested 6-year-olds with pre-subject *only* sentences in which the subject occurred after the finite verb in sentence final position (OVS), which is possible in German due to its relatively free word order (e.g. *Den Ballon hat nur die Maus*, literally: the_acc_ balloon has only the mouse, meaning: “Only the mouse has the balloon”). The crucial point here is that *only* unambiguously takes scope over the subject in this position. Nevertheless, the children again showed an asymmetrical pattern with better performance for pre-object compared to pre-subject *only* sentences. This led Müller and colleagues to argue against a syntactic explanation for this asymmetrical performance and to propose an approach that considers the typical convergence between information status and grammatical function. In many languages, including German, the subject is usually associated to the topic while the canonical focus position is the direct object, which sits in the nuclear stress position [2, 26]. Thus, Müller et al. assume that children adhere to a preference of assigning topic-hood to the subject, which conflicts with the focus status that is necessary to associate the focus particle to the subject. Therefore, children are unable to interpret the pre-subject *only*. As the pictures used in their task also always matched the sentences without an FP, accepting the sentence would be the resulting pattern. Most importantly, this hypothesis assumes that the correct (i.e. adult-like) syntactic representation is available to the children but that the mismatch between syntactic information and pragmatic principles leads children to arrive at an incorrect interpretation.

In contrast to the syntactic proposal by Crain and colleagues, Müller et al.’s account states that children must initially parse the pre-subject *only* sentences correctly because otherwise they should not take the FP as being associated to the sentence subject into account and no conflict would arise. This aspect of the hypothesis can only be tested by a method that sheds light on the ongoing interpretation process before a decision is made. Therefore, we conducted an eye tracking study using the visual world paradigm. So far, studies comparing children’s comprehension of unambiguous sentences with pre-subject and pre-object *only* have used experimental methods that do not tap into the processing of these sentences but only reveal the final interpretation that children select for the sentence in a specific experimental setup. In the visual world paradigm [27, 28] visual information serves as a frame of reference for spoken language input. By controlling both sources of information–and assuming specific linking mechanisms [29]–visual attention can be interpreted as a marker of parsing decisions and sentence comprehension. Moreover, it is possible to combine this paradigm with an instruction so that in addition to the eye movements explicit responses can be analyzed. Previous studies using eye tracking within the visual world paradigm have revealed that this method is also very suited to uncovering aspects of children’s ongoing processing of information provided by a sentence [30–32], so that this method lends itself to comparing children with adults. Furthermore, previous research has also shown that children and adults fixate visual information that is not directly mentioned in the sentence but constitutes contrastive information to the sentence focus [31, 33]. This makes the eye tracking method especially suitable for the purpose of our study.

We tested the hypothesis that German-speaking 4-year-old children consider an adult-like initial parse of the pre-subject *only* sentences in an eye tracking study. To this end we compared the looking patterns during the processing of sentences with a pre-subject or pre-object *only*, or without an FP. This paradigm is especially useful in our study because visual information, which is not mentioned, is relevant for evaluating the truth of the sentence. Upon hearing a pre-subject *only* sentence like *Only the elephant has a kite*, it is necessary to check whether other characters in the display also have a kite in order to evaluate the sentence. Thus, enhanced visual attention (i.e. eye gazes) to the characters that represent the subject alternatives (i.e. the subject alternative set) is expected if the sentences with pre-subject *only* are initially parsed correctly.

In Study 1, we assessed the looking patterns of adults and children. Adult participants were included to ensure that the expected looking pattern indeed holds, which then can be used to evaluate children’s performance. As part of the experiment, participants further had to decide whether the sentence was a match to the picture or not, allowing for an assessment of the final interpretation that the children assigned to the sentences. We made the following predictions: the proportion of looks to the subject alternative set is higher in adults for sentences with *only* in pre-subject position than in pre-object position or for sentences without *only*. In sentences with *only* in pre-subject position, the need to verify the proposition (‘having a kite’ in the example above) for each of the non-mentioned characters will result in a high proportion of looks. However, if the focus particle is *not* in pre-subject position, then no subject alternative set is construed and therefore it will attract only few looks (if any). For children we expect a similar looking pattern. Assuming that the difficulties that 4-year-old children have with pre-subject *only* in offline tasks are not syntactic in nature, an implicit measure like eye tracking will reveal an adult-like performance. A qualitatively different looking behavior in children compared to adults would imply deviant processing of these structures already at the lexical and syntactic levels. In contrast to the eye movements, we predict a difference between children and adults in the offline responses. Here, children need to evaluate (by saying *yes* or *no*) whether the spoken sentence is true in regard to the picture. Based on previous findings by Müller et al. [17], accuracy in adults will be at ceiling for all sentence types while children’s performance will vary gradually: relatively high accuracy for sentences without *only*, lower values for pre-object *only* and yet lower scores for pre-subject *only*.

Since adults do not show problems with sentences containing *only* in different positions, their eye gaze patterns serve as a standard for interpreting the children’s gaze patterns. This is crucial, as we will argue that this implicit measure reflects processing of linguistic structures that is unaffected by any additional operations that overt responses may require.

## Study 1

Both studies described in this article have been approved by the Ethics Board of the University of Potsdam (approval no 14/2010).

### Method

#### Participants

Seventeen children (10 girls) with a mean age of 4.5 years (range: 4.0 to 4.8) participated in Study 1. All children were raised monolingually with German as their native language and were typically developing according to the information obtained from their parents. They had no known visual or auditory deficits.

Data from two additional children had to be excluded because of inattention during the eye tracking session. We tested 17 adults (16 female) with a mean age of 21.9 years (range: 19–31) as controls. All of them were students at the University of Potsdam and native speakers of German. Data from two other adult participants were discarded due to technical difficulties with the eye tracker.

#### Materials and design

We used the pictures and audio material from the sentence-picture verification task developed by Müller et al. [17]. Minor adjustments were necessary for their use in an eye tracking study: resizing of the images, adjusting of the objects and object positions within them, and changing of the length of the pauses in the sound files.

**Fig 1:**
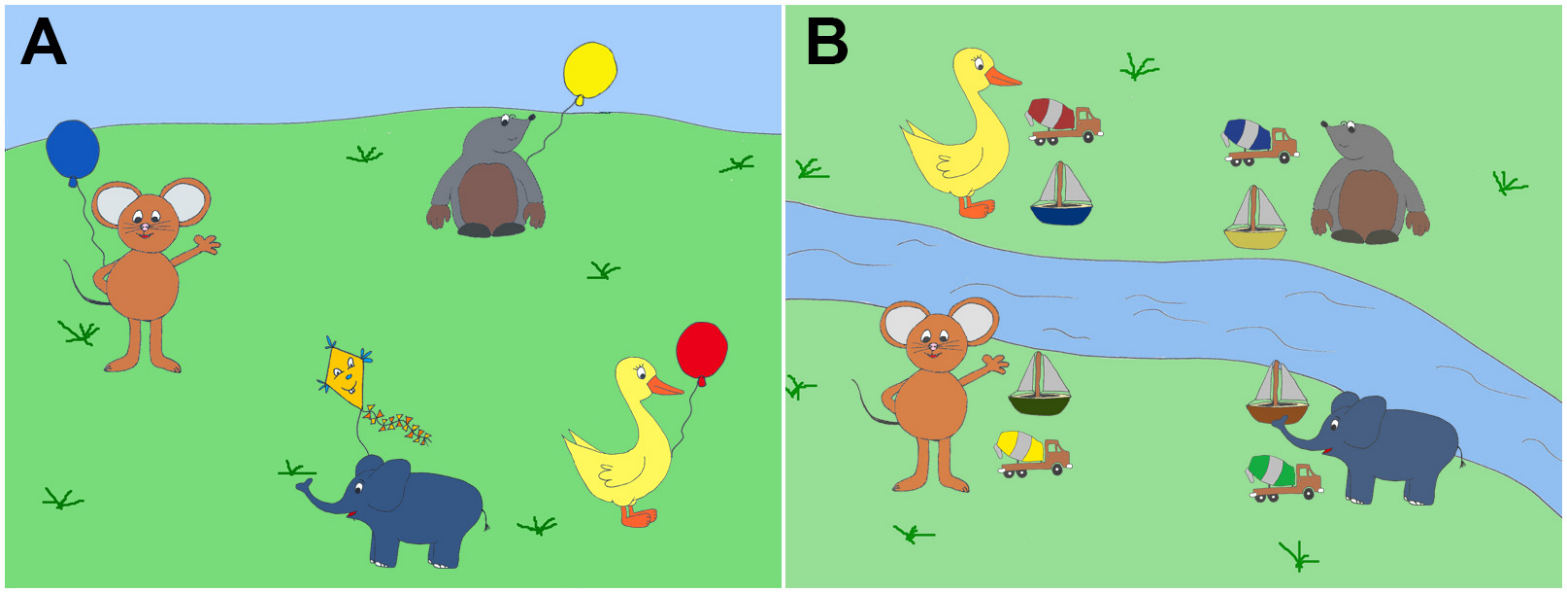
Two examples of the visual displays. (A) Scenario for the test sentence *Only the elephant has a kite* (expected answer: yes). (B) Scenario for *The duck has only a boat* (expected answer: no). The images are for illustrative purposes only; They are very similar but not identical to the ones used in the experiment.

Each image depicted four cartoon characters (mouse, elephant, mole, duck) from a popular German children’s TV show. The spatial position of the characters varied among the images (as did the background) but was balanced so that each character appeared equally often in every part of the images (left/right and top/bottom). Each character possessed one or two items (Fig 1) which were located close to it.

The target character was always the subject of the sentence, e.g. the elephant in *Only the elephant has a kite*. Each of the four characters appeared equally often as the target. In order to make sentences true or false, the distribution of items in the image had to be varied across the conditions. For pre-subject *only* sentences all characters possessed only one item each. In true descriptions, this item was unique for the target character and different for the non-target characters while it was the same for all characters in false descriptions. For pre-object *only* sentences, the non-target characters were always depicted with two different items each. The number of items of the target character depended on the status of the item as intending to elicit a *yes* or a *no* response. The target character possessed only one item in the *yes*-condition but two in the *no-*condition. For sentences without an FP, each character possessed a completely different item. The target character’s item was mentioned in true descriptions while in false ones some other, not depicted item was mentioned. Two versions of each image (using the same background and positioning of the characters but with different items) were created to control for image-specific effects for the conditions with *only* such that they appeared once with a pre-subject *only* sentence and once with a pre-object *only* sentence. The images of the NoFP condition remained unchanged and were repeated once.

Auditory stimuli consisted of pre-recorded introductions and test sentences for each image. The introduction mentioned the three non-target characters and their items, thereby motivating the use of the focus particle *only* by establishing a common ground that included all characters and objects. The test sentence stated that the target character possessed an item X in one of three conditions: without *only* (NoFP), with *only* in pre-subject (Pre-subj) position, or with *only* in pre-object (Pre-obj) position. Depending on the pictorial information, this statement was either true or false. An example of an auditory stimulus (Fig 1A) is given in (2) below.

(2) Die Maus, der Maulwurf und die Ente haben einen Ballon.

The mouse, the mole, and the duck have a balloon.

Nur der Elefant hat einen Drachen.

Only the elephant has a kite.

All sentences were recorded by a female native speaker of German. She was instructed to produce the stimuli in a child-directed manner with natural stress. This resulted in prosodic differences between the sentences: in Pre-subj sentences main stress fell on the subject, in Pre-obj sentences on the FP, and in NoFP sentences on the direct object. The intensity of all sound files was normalized to 70 dB using Praat [34].

The images and sound files were combined into video clips for presentation on the eye tracker. We created 48 video trials, which were shown to each participant. The numbers were balanced for the three sentence types (16 trials each) and for the response type within each sentence type. Due to differences in the number of mentioned items, the duration of the introduction varied across trials. As a result and in order to keep the speech rate natural, the onset of the test sentences within the video-sequences varied across trials. The timing of a video was as follows: (1) presentation of the picture in silence for 1 s, (2) introductory sentence between 2.5 and 5.5 s, (3) pause of about .7 s, (4) test sentence of about 1.5 s, and (5) silence for 5 to 6 s. For each trial, the pauses were adjusted to set the video length to 11 or 12 s. The silence period at the end was included to allow eye gazes to be analyzed after the sentence presentation was finished. As the final word of the sentence (i.e. the direct object) was necessary for sentence interpretation, it was expected that eye movements resulting from parsing and interpretation processes would also occur in a time window that extended beyond the end of the acoustic stimulus.

#### Apparatus and procedure

Stimulus presentation and data acquisition were carried out with ClearView (version 2.5.1) on a Tobii 1750 binocular corneal reflection eye tracker. This system tracks gaze positions every 20 ms with a spatial accuracy of .5 to 1° and a recovery time after track loss of about 100 ms. Only valid data was analyzed, i.e. when at least one eye could be correctly tracked.

Participants sat in a lean-back chair in a dimly lit room with their eyes at a distance of about 60 cm from a 17 inch (1280 x 1024) TFT display. All visual stimuli had a resolution of 800 by 600 pixels subtending a horizontal viewing angle of 19.9° and a vertical one of 15.0°. The background screen color was set to black. The system was calibrated to the participant’s eyes with a 5-point automatic calibration using a red dot on a black background.

After obtaining written informed consent from the participant or in the case of children the parents, the participants were accompanied to the room with the eye tracker. After seating the participant and adjusting sitting position and eye tracker, it was checked that the recognition of the pupils by the eye tracker was central in a virtual box of about 30 by 16 cm. Then, the calibration procedure was started. In case of suboptimal calibration results the procedure was repeated up to three times. The experiment was started when the spatial precision of the gaze for each calibration position was classified as adequate by the system and/or the experimenter.

The experiment consisted of two blocks with 24 trials each. The first block required no verbal response while for the second block participants had to give a *yes* or *no* response depending on whether they judged the sentence as matching the visually presented scenario or not. The response was to be given after the completion of the trial (indicated by an acoustic signal following the silence period of each trial). The final frame of the image remained on the screen until the response was noted and the child was ready for the next trial. This blocked procedure was a precaution because in a study by Brandt-Kobele and Höhle [35] an additional task (in their study: pointing) reduced the effects from the eye gaze data compared to a listening only condition. In order to keep the trials comparable between the blocks (i.e. an equal silence period after the sentence presentation) we opted for a short delay of the response.

Each participant viewed the same 48 videos. We pseudo-randomized the order within each block using the following restrictions: no repetition of the same target character position in two consecutive trials, a maximum of two consecutive trials of the same sentence type, at most one repetition of the same target character, a maximum of three trials in a row containing the FP, and an equal amount of expected *yes/no* answers within each block. To control for order effects we compiled two lists in which the presentation order was switched between block 1 and 2.

Practice trials preceded each block and displayed only one character with one item, e.g. *The mouse has a cup*. Two practice trials (both true) preceding the first block served to establish that an item next to a character belonged to it. Four practice trials (half of them true) at the beginning of the second block were included to encourage both *yes* and *no* responses. For the first block, participants were instructed just to listen to the sentences and to look at the pictures. Before starting the second block, participants were instructed to say whether the sentence matched the picture or not. For the children a cover story explained that the speaker was still learning German and the child’s response would help her to learn. No feedback was given during the experimental trials.

The trials were presented in groups of three without pauses between them. After each group an attention-getting stimulus with an animated cartoon character (e.g. Snoopy, Kitty, Elmo)–unrelated to the characters used in the study–was presented for as long as the experimenter considered appropriate. This also allowed the participants to re-focus attention on the screen or, if necessary, to adjust the sitting position. A testing session lasted about 20 to 25 minutes.

### Results

#### Sentence-picture verification

**Fig 2:**
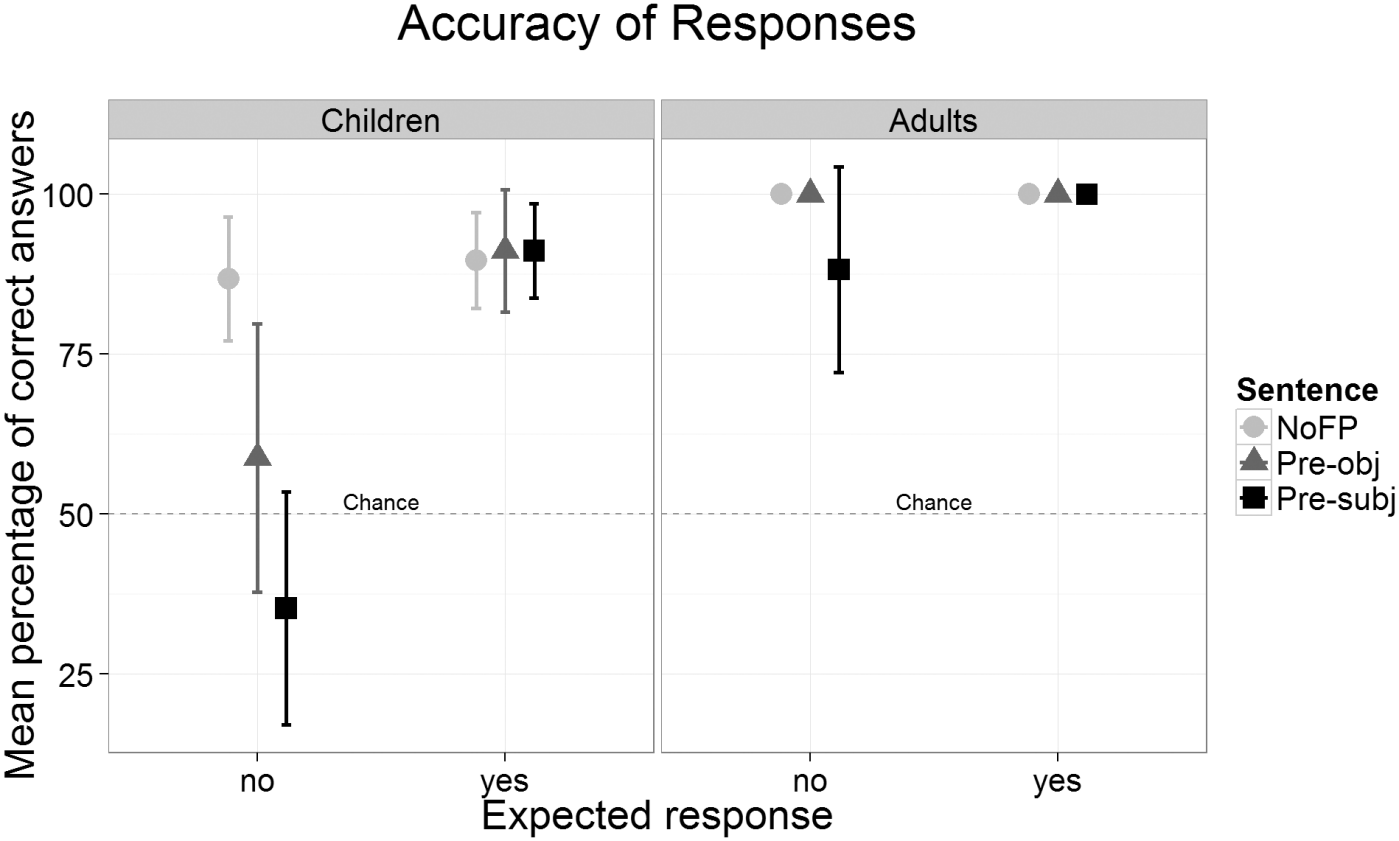
Mean accuracy scores for each sentence type and expected response in children and adults. Error bars denote two standard errors.

The aggregated accuracy scores from the second block are shown in Fig 2. Four responses could be given by each participant for each condition (sentence type and expected response). Adults’ performance was at ceiling (100% correct) for the Pre-obj and the NoFP sentences but decreased to 88.2% correct responses for Pre-subj sentences with expected *no* responses. This lower overall performance was due to two participants who consistently responded incorrectly in this condition. However, there was no significant difference between Pre-subj and Pre-obj sentences (*t*(16) = 1.46, *p* = .164). Overall, there was no significant difference between the number of correct *yes* and *no* responses (100% vs. 96.1%, *t*(16) = 1.46, *p* = .164).

Children responded correctly significantly more often with expected *yes* responses (i.e. for matching pictures) compared to the expected *no* responses (91% vs. 60%, *t*(16) = 3.62, *p* < .01). For the expected *yes* responses there are no accuracy differences between the three sentence types (NoFP: 89.7%, Pre-obj: 91.2%, Pre-subj: 91.2%, all *t*<1, all *p*>.57). For expected *no* responses, accuracy varies with sentence type. It is higher in NoFP compared to Pre-obj sentences (86.8% vs. 58.8%, *t*(16) = 2.51, *p*< .05) and also higher in Pre-obj compared to Pre-subj sentences (58.8% vs. 35.3%, *t*(16) = 2.70, *p*< .05). With these latter two sentence types children’s performance did not exceed the chance level (Pre-obj: *t*(16)<1, *p* = .413; Pre-subj: *t*(16) = 1.61, *p* = .126). These group means disguise the individual response behavior, which was quite consistent and not random. Counting the children who gave at least 75% correct *no* responses to the FP sentences (passers) yielded 11 Pre-obj passers and 6 Pre-subj passers, again showing that performance is higher in Pre-obj than in Pre-subj sentences (of the 6 Pre-obj non-passers there was only a single correct response. Of the 11 Pre-subj non-passers 8 never gave a correct response, one child gave one correct response (25%) and two children gave two (50%). Thus, chance level performance at the group level does not necessarily reflect guessing behavior at the individual level.

#### Eye gaze data

Data points for which the eye tracker could not determine the gaze position for at least one eye were removed (15% of all data in children, 7% in adults). For the final analysis only trials with less than 50% track loss were kept. Many papers on eye tracking studies with infants and children report this 50% criterion. The track loss per trial in this study was on average 12.7% for children (SD = 21.2, min = 0, max = 100), and 6.2% (SD = 6.3, min = 0, max = 50.0) for adults. For children this resulted in 44.6 trials on average out of the 48 trials, and for adults 46.5 trials.

**Fig 3:**
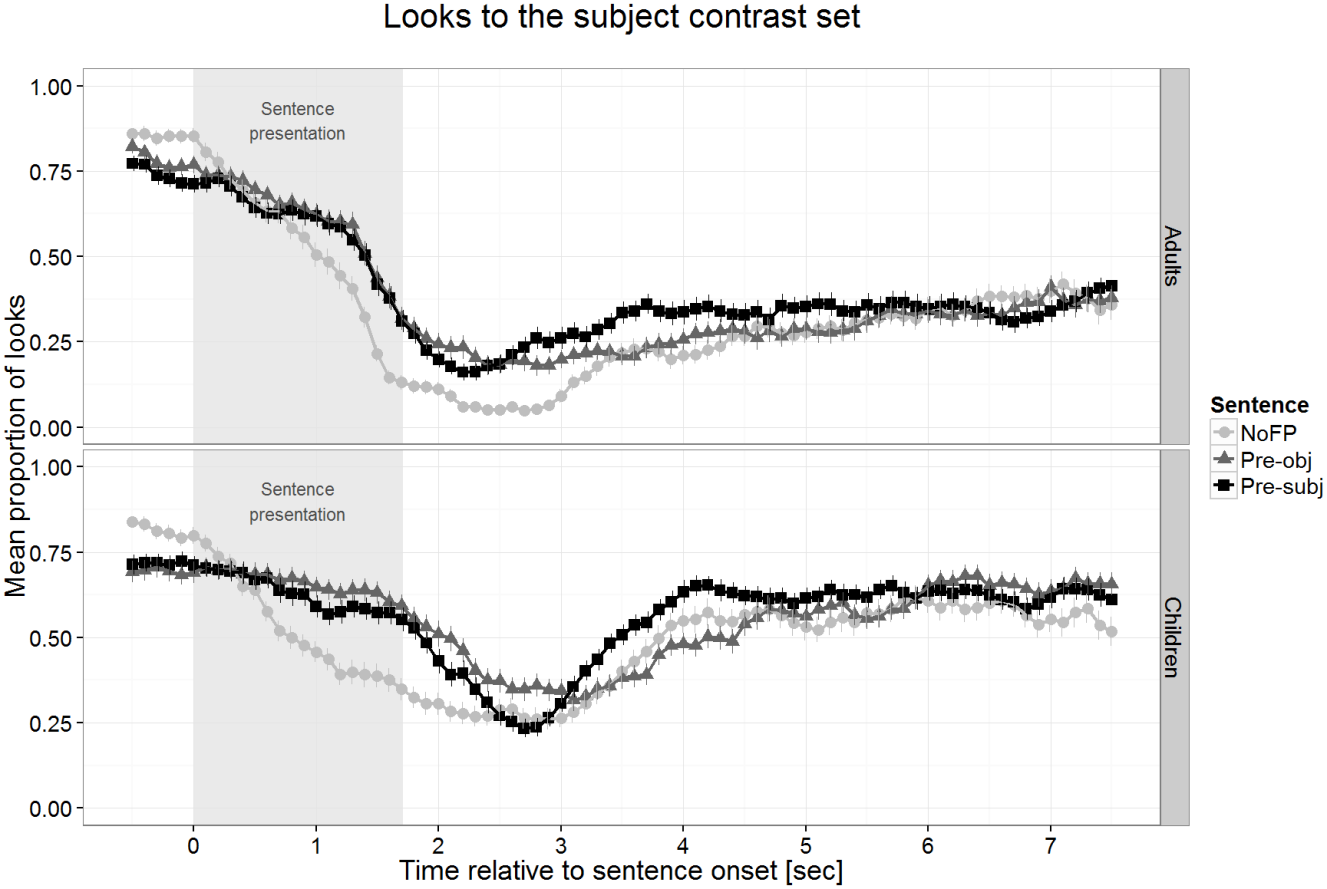
Looks to the subject alternative set by children and adults. Data shown are averaged over both testing blocks.

We created four equally sized (about 62,000 square pixels) spatial areas of interest (AoIs), one for each character including its items. All gaze positions were classified as being in one of the four AoIs or not. The three AoIs of the non-target characters were combined into a single AoI as they form the subject alternative set. The looks to this AoI served as the dependent variable. The averaging procedure (first across bins of 100 ms within each trial and participant, then across trials for each sentence type within participants, and finally the grand average) created a proportion of looks to the subject alternative set with values between zero and one. The difference between one and the value of the proportion of looks to the subject alternative set were looks to the target character and, to a much lesser degree, looks outside all AoIs. Fig 3 displays the time course of looks to the subject alternative set, i.e. the characters *not* mentioned in the test sentence for both participant groups.

Looking proportions to the subject alternative set started out at about .75, which is predicted by chance as this set comprises three of the four characters in the display. With the sentence onset looks shifted away from the alternative set (more or less quickly) and reached a minimum after roughly two to three seconds, before looking proportions to the subject alternative set seemed to increase again. This increase varied between the sentence types. To assess these differences statistically, we divided the time axis into windows of one second (Table 1). For each window, we compared both the NoFP and the Pre-obj sentences to the Pre-subj sentences in a linear mixed-effects model. We modeled the looks to the subject alternative set for each time window separately including the within-participant factors Sentence Type, Block, and Expected Response (yes/no) and the between-participant factor age using the library lme4 [36] for R [37]. The formula and the detailed output are given in the table in S1 Table.

**Table 1:** Mean proportions of looks to the subject alternative set.

Age group	Sentence	0	1	2	3	4	5	6	7
Children	Pre-subj	.675	.563	.313	.476	.628	.630	.626	.639
Children	Pre-obj	.689	.611	.404	.378*	.529***	.578**	.662	.627
Children	NoFP	.642	.375***	.276*	.385**	.564***	.561***	.592***	.563***
Adults	Pre-subj	.669	.449	.200	.314	.338	.355	.336	.408
Adults	Pre-obj	.701	.456	.201	.221*	.275***	.305**	.343	.379
Adults	NoFP	.692	.285***	.065*	.188**	.266***	.309***	.364	.380***

The proportions are averaged across blocks and expected responses for each time window after the onset of the sentence. Each window is one second long; window 0 starts with the onset of the test sentence. Significant differences compared to the Pre-subj sentences are marked by asterisks:

*: *p* < .05,

**: *p* < .01,

***: *p* < .001.

**Fig 4:**
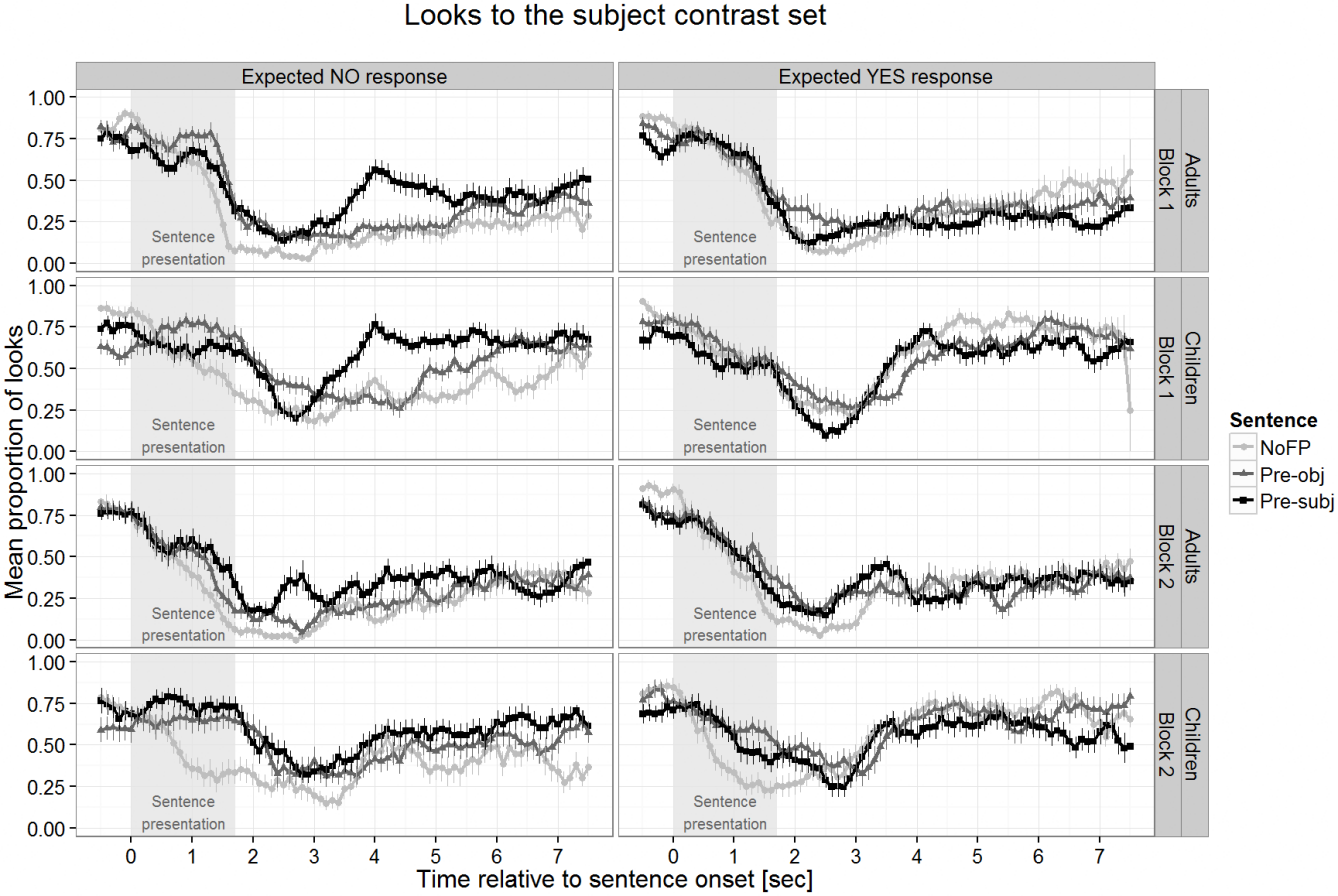
Looks to the subject alternative separated by testing block and the type of expected response.

Children’s data in Pre-subj sentences served as a baseline to which the other sentence conditions were compared. Differences in adults’ performance compared to children’s show up as interactions of age with sentence type–more accurately, the sign of the interaction needs to go in the opposite direction, otherwise the interaction shows the same effect in adults as in children, just to a stronger degree (if this is the case it is stated). Apart from non-significant interactions with age, which show that there is no difference between children and adults, we report only significant results. The p-values were calculated using the R library lmerTest (http://cran.r-project.org/web/packages/lmerTest). The effects of age, block, and expected response were only considered if they interacted with sentence type, i.e. when they modulated the difference between the sentence types, because overall effects of these factors were not of interest for our research question. Fig 4 below plots the data separated by block. Let us first look at the comparison in looking proportions to the subject alternative set between Pre-subj and Pre-obj sentences. For children, these proportions were significantly higher for Pre-subj in windows 3 to 5 (*t* = 2.44, *p* < .05; *t* = 4.75, *p* < .001; *t* = 2.78, *p* < .01, respectively), showing that the position of the FP *only* had an effect on the visual focus of children. The same looking pattern was present in adults as there is no interaction with age in these windows (all *t*<1, all *p*>.48). The difference between Pre-subj and Pre-obj sentences was modulated by Block (in window 4: *t* = 2.62, *p* < .01) and expected response (in windows 4 and 5: *t* = 3.80, *p* < .001 and *t* = 2.36, *p* < .05) such that it was present mainly in the first block and for the trials requiring a *no* response. In adults the same effects of block and expected response were found (no interaction with age: all *t*<1, *p*>.37). The second comparison concerned the difference between Pre-subj and NoFP sentences. Children’s looking proportions were higher for Pre-subj sentences in windows 1 to 7 (all *t*>2.64, all *p* < .05). For adults, a highly similar pattern was observed for windows 1 to 5 and 7 (all *t*<1.52, all *p*>.13) but not for window 6 (*t* = 2.66, *p* < .01). This effect was modulated in the same way as in the previous comparison by block (windows 1 and 4: both *t>*2.06, both *p <* .05) and expected response (windows 1 to 7: all *t>*2.14, all *p <* .05). The effects were present mainly in the *no* response trials of block 1. As before, the same pattern was observed for adults (no interactions for windows 1 to 5 and 7: all *t*<1.87, all *p*>.06); only in window 6 was the effect of expected response the opposite of that in children (*t* = 2.41, *p* < .05).

To summarize, the expected looking pattern of more looks to the subject contrast for Pre-subj sentences was found in windows 3 to 5 when compared to Pre-obj sentences and in windows 1 to 7 when compared to NoFP sentences. For both sentence types, the effect was primarily present in the first block and for trials with an expected *no* response.

### Discussion of Study 1

Overall, our findings reveal a discrepancy between the results from the eye tracking study and the results from the task in which the children had to decide about the match between picture and sentence. While adults–unsurprisingly–almost always answered correctly in the sentence-picture verification, and children were also at ceiling in trials requiring a *yes* response, as a group children showed chance level performance in the pre-subject and pre-object *only* conditions. But still, the number of correct *no* responses was larger for pre-object compared to pre-subject *only* sentences. This confirms our hypothesis and replicates findings from Müller et al. [17], who also observed this pattern in 4- and 6-year-old children. In contrast to the differences between children and adults in response accuracy in the verification task, eye gaze patterns are highly similar across the two groups. In line with our prediction, we observed an increase in looks to the subject alternative set upon hearing Pre-subj sentences relative to Pre-obj sentences or sentences without an FP. In the following, we will first discuss the results from the picture-sentence verification task and those from the eye tracking study separately, and then try to integrate them.

First of all, the results from the sentence-picture verification show a difference between the responses that were correct acceptances of the sentence as matching the picture compared to the responses that were correct rejections of a sentence that did not match the picture. Only the latter revealed that children still struggled with the sentences containing an FP and that there is an asymmetrical performance between pre-subject and pre-object *only* sentences. This effect of the expected response emphasizes that the *yes* responses for matching sentences have only a limited value as evidence for a correct sentence interpretation in this task. Remember that the sentence always matched the visually presented scenario (in both expected response conditions) if the focus particle was ignored. Thus, the correct *yes* responses do not necessarily show that the FP was integrated into the interpretation of the sentences, but rather only the correct *no* responses reveal its correct interpretation. Therefore, our discussion will focus on these correct rejections.

According to the correct *no* responses given in the verification task, 4-year-olds have problems interpreting sentences containing the FP *only* in an adult-like fashion, which is in line with a number of previous findings [5–7, 11, 17]. Furthermore, the better performance with pre-object *only* also replicates previous findings that showed that children are more inclined to associate the FP to the sentence object or the VP than to the sentence subject [7, 9, 10, 13, 17, 20, 21]. However, while Müller et al. [17] found chance level performance for pre-subject *only* sentences and above chance for pre-object *only* sentences in the *no* responses of their 4-year-old participants, the children tested in this study were at chance level in both pre-subject and pre-object *only* sentences. However, the performance pattern is similar across the two studies, with significant better performance and a higher number of children reaching an at least 75% correct criterion in the *no* responses for the pre-object as compared to the pre-subject sentences. The overall lower accuracy rates in the present study might be attributed to procedural and/or individual differences. The procedure in Müller et al. was shorter (24 trials instead of 48) and involved direct interaction with the experimenter as well as a hand puppet. This might have had a beneficial effect on the outcome as it was more engaging and rewarding.

Turning to the eye gaze data, the patterns of children show that they allocate their visual attention to the subject alternative set according to the presence and position of the FP in the sentence in very much the same way as adults do. This is evidence that 4-year-old children are not only able to detect the presence of the particle in the sentence but also seem to check the visual information depending on the position of the FP, specifically and most importantly paying more visual attention to the characters that form the subject alternative set after hearing a non-matching sentence with pre-subject *only* as compared to a sentence with pre-object *only* or without an FP. As the increased looking proportions to the subject alternative set–both in children and adults–only occurred after the sentence presentation was completed, we assume that this results from the process of evaluating the match of the sentence interpretation to the visually presented information. During this process a representation of the sentence interpretation must guide the visual attention to the display as revealed by the differences in looking patterns across the sentence conditions. The fact that the subject alternative set attracts more visual attention after the presentation of a pre-subject *only* sentence strongly suggests that listeners (including children) initially parse the sentences correctly and generate a representation of the sentence meaning that includes the information provided by the occurrence and the position of the FP.

In our data, this effect was primarily observed in the first block of the experiment and for trials with an expected *no* response. At this point, we can only speculate about the conditions that led to this restriction. Remember that the first block included no task while in the second block participants had to decide explicitly on the match between sentence and picture by giving a *yes* or a *no* response. The fact that the eye gaze patterns were stronger in the first block parallels findings by Brandt-Kobele and Höhle [35], who also found more pronounced eye gaze patterns in a looking-only procedure compared to a procedure in which the children had to select one of the presented pictures as matching a sentence. They suggested that children may visually check the whole display more often if they have to give an explicit answer–an explanation that may also be transferred to our findings. In addition, the presence of a task was confounded with the position of the block that required the explicit verification in the present study. As this block was always presented in the second half of the experiment, we cannot rule out that a general decrease in attention led to less pronounced eye gaze patterns.

The influence of the expected response on the eye gaze patterns suggests that the verification for non-matching sentence-picture pairs (i.e. expected *no* response trials) proceeds differently than for matching pairs. While this should not be the case in principle (the sentence is identical regardless of the image information), it could be related to the way the trials were designed in this study. Remember that prior to the test sentence all the characters except the target character were verbally introduced while the visual display was already presented. The fact that the visual displays for the pre-subject *only* and the pre-object *only* sentences were different with respect to the number of items possessed by the characters might have created an expectation about the following sentence. If this expectation was not met, a more intense (compared to the situation in which the expectation was met) re-check of the visual information on the display might have been initiated, leading to the more pronounced gaze pattern if the expected answer was a *no* response.

As these limitations are completely parallel in children’s and adults’ data, we do not think that they contradict our interpretation of the results as showing specifically enhanced visual attention to the subject alternative set after hearing a sentence with pre-subject *only* and thus as providing evidence for an initial correct parse of the sentence by children. If this interpretation is valid, the main question that emerges from this study is why children’s performance in the verification task does not reflect the correct sentence interpretation, or to put it in other words: what is the source of the discrepancy between the adult-like pattern in the eye tracking and the low performance for the pre-subject *only* sentences in the verification task? This study is not the first one showing such a discrepancy. For example, Brandt-Kobele and Höhle [35] found chance level performance in a task that tested 4-year-olds’ ability to exploit the information provided by verb inflection as a cue to subject number when the children had to select from two pictures by pointing to the matching one. However, eye tracking data collected with the same task revealed significantly increased looking proportions to the matching picture. More similar to our data, Zhou and colleagues [31] showed that Mandarin-learning 4- to 5-year-old children’s gazes were directed to the visually presented alternative set of either the modifier or the head noun in a construction like *Only John’s apple is red* depending on the placement of a focus accent. However, when the match between the sentence and the visual information had to be judged, children’s responses revealed a constant association of the FP to the modifier while adults shifted between a modifier and a head noun association depending on the accent placement. These results suggest that visual attention may be a more direct and reliable indicator of children’s linguistic processing and thereby their linguistic abilities than dependent measures which rely on children’s decisions about the match or mismatch between a linguistic and a visual stimulus.

One of the major differences between looking to a visual display while listening to a sentence and making an explicit decision about their match lies in the impact that cognitive skills beyond linguistic ones have on the outcome measure. It is likely that directing one’s eye gaze is less affected by additional cognitive skills than providing an overt *yes* or *no* response as a result of matching different kinds of representations, evaluating their fit, and making a decision about this fit.

More specifically, we assume that general cognitive abilities related to the maturation of so-called cognitive control (or executive functions) might play a role here. Cognitive control encompasses higher order cognitive processes like inhibitory control, working memory, and attentional flexibility and is considered to mature relatively late during childhood (for a developmental review, see Hughes [38]). Only recently has the development of cognitive control been considered as a potential cause for some aspects of children’s non-adult-like performance in sentence comprehension. Novick, Trueswell, and Thompson-Schill [39] have discussed children’s immature cognitive control as a reason for their non-adult-like performance with garden-path sentences which require a reanalysis of the initially assigned structure like in sentences such as *Put the frog on the napkin into the box*, in which children have been shown to interpret the first PP as the goal of the action while adults quickly revise this interpretation upon hearing the second PP [30]. Novick and colleagues [39] assume that children develop an automatic parsing analysis based on the distribution of specific linguistic elements (in this case the fact that the verb *put* is frequently followed by a PP denoting the goal of the action). In their approach cognitive control processes are more strongly involved if a less dominant syntactic analysis has to be pursued.

So far, direct evidence of a relation of the development of cognitive control to the development of sentence comprehension is scarce. In a recent study, Minai, Jincho, Yamane, and Mazuka [40] report that children’s level of cognitive control–in this case the ability to inhibit salient visual information–is related to their comprehension of sentences with the universal quantifier *every*, which has been shown to be difficult for children in a number of previous studies [20, 41, 42].

We propose that immature cognitive control may also play a role in children’s selection of an interpretation for pre-subject *only* sentences. As mentioned above, the default position of focus is considered to be the most deeply embedded constituent in a sentence, which typically is the sentence object. Following the reasoning by Novick et al. [39], we argue that this default affects the comprehension of a sentence with pre-subject *only* by creating competition between the actual focus assignment and the default, which has to be overcome during the interpretation process. According to this assumption, children’s ability to inhibit the dominant (i.e. default) interpretation, which would take the VP as the locus of the focused constituent, should selectively be related to their correct interpretation of sentences with pre-subject *only*. A second component of cognitive control that may affect the outcome of the verification task is working memory, as the sentence interpretation has to be kept in memory during the process of matching the linguistic and visual information. However, working memory capacity should be associated to the performance with both pre-subject and pre-object *only* sentences. To test the relations of cognitive control to the performance in the picture-sentence verification, we re-ran the experiment from Study 1 with a different group of 4-year-olds and included the flanker task [43] as a measure of inhibitory control as well as the forward digit span as a working memory estimate [44] in Study 2.

## Study 2

### Method

Participants. The final sample in Study 2 comprised 34 4-year-old children (18 girls) with a mean age of 4.5 years (ranging from 4.1 to 4.9). Data from one additional girl had to be removed due to technical failure during the eye tracking part. All tested children were raised monolingually in German and were typically developing according to the parent questionnaires without any visual or auditory deficits. None of the children had already participated in Study 1.

#### Materials and design

Visual and auditory material for the sentence-picture verification were identical to Study 1.

We modeled the flanker task [43] after the attentional network test for children by Rueda and colleagues [45], a test also appropriate for 4-year-old children [46] which uses colored line drawings of fish facing left or right and is embedded in a game about feeding the fish.

**Fig 5:**
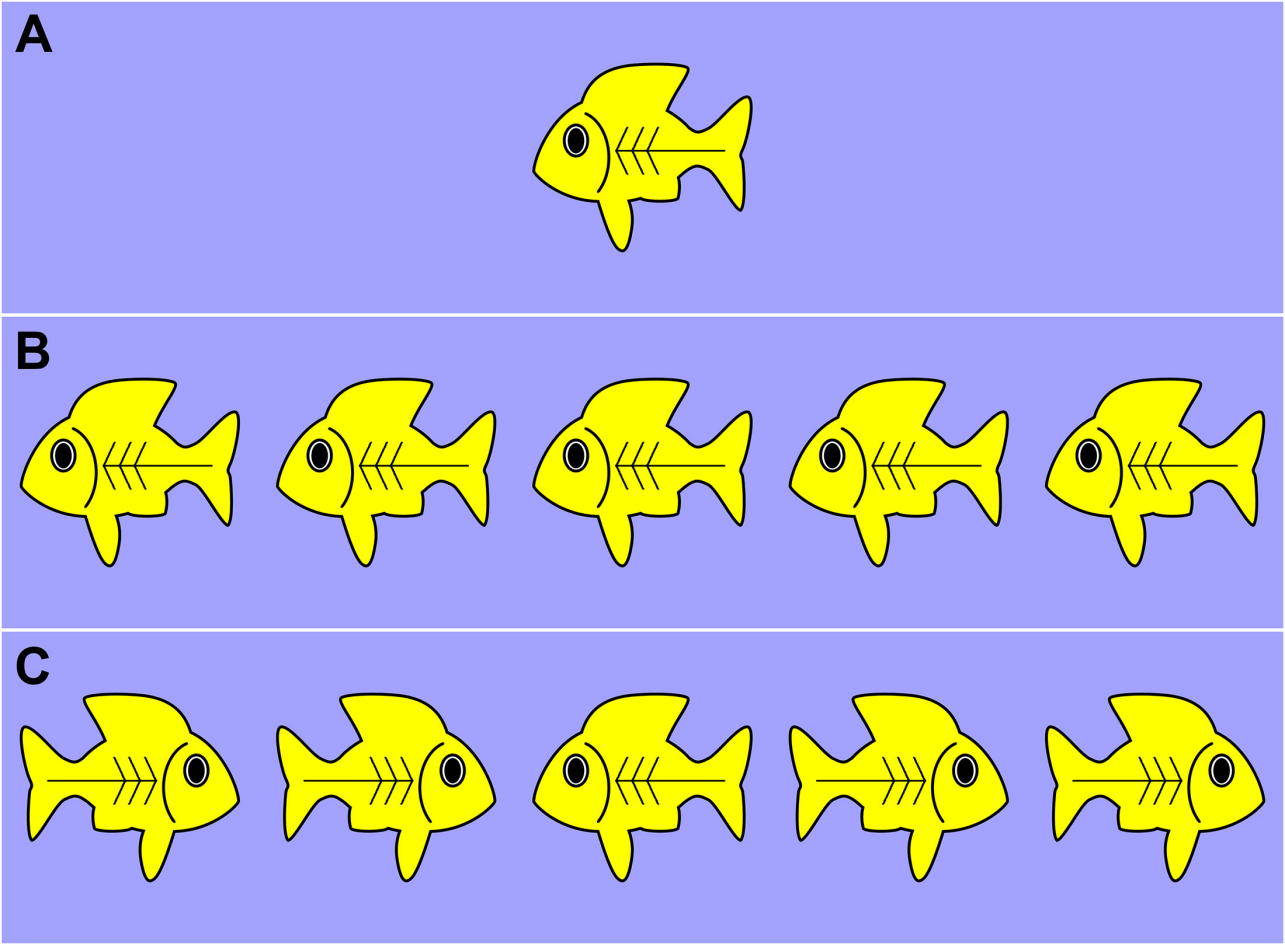
Examples of the three conditions in the flanker task. The target fish is always the one central on the screen. (A) Neutral condition without flankers. (B) Congruent condition: flanking fish face the same way as the target fish. (C) Incongruent condition: the direction of the flanking fish conflicts with the direction of the target fish. These examples show left-facing target fish, but there were right-facing ones as well. The images are for illustrative purposes only; They are very similar but not identical to the ones used in the experiment.

The task is to press a button as quickly as possible on the side that the fish in the middle, the target fish, is facing. There were three conditions (Fig 5): presentation of a single fish (neutral condition), and presentation of a target fish flanked by two other fish on each side, which either face in the same direction as the fish in the middle (congruent) or do not (incongruent). All fish were identical in size and colored in yellow. Two stimuli were created for each of the three conditions, one with a left-facing target fish, the other with a right-facing one. These six different stimuli were repeated in eight blocks, resulting in 16 trials per condition.

The flanker effect, i.e. the difference between congruent and neutral trials, can be considered as an effect of additional information or noise. Of interest for our purposes is the inhibition effect, i.e. the difference between the incongruent and the congruent condition, an index of the ability to focus on the relevant information and to suppress interfering stimuli. This effect is marked by an increase in error rate and response latency. A larger interference effect indicates lower inhibitory control abilities.

We will use the interference effect measured by response times (RT_incongruent_−RT_congruent_) as an individual diagnostic of the cognitive control ability. Children with more advanced cognitive control should be less affected by conflicting information so that their individual interference effect should be smaller.

The forward digit span was assessed in the commonly used way [47, 48]. Children were presented with sequences of pseudo-randomized digits at a rate of one per second, which they were required to repeat in the same order.

#### Apparatus and procedure

Each test session consisted of the eye tracking part with the sentence-picture verification task, the flanker task, and the forward digit span test at the end.

The apparatus and procedure including the eye tracking setup for the sentence-picture verification were the same as in Study 1 with two changes. First, we asked for explicit responses in *all* trials from the beginning in Study 2. Moreover, we encouraged participants to respond as quickly as possible. The instruction for explicit responses as well as the four practice trials that preceded the second block in Study 1 were presented at the beginning of the experiment in Study 2.

Presentation and data collection of the flanker task were administered with DMDX [49] on a notebook using two big colorful response buttons that were placed in front of the child. The DMDX software logged the response side together with the reaction time. The display had a resolution of 1280 by 800 pixels and the background color was set to dark blue. Test items were presented in eight blocks. Each block consisted of six trials, two of each condition. The direction (left/right) of the central target fish was counterbalanced within each condition within each block. The presentation within a block was randomized by DMDX such that a maximum of three trials in a row required the same response and at most two consecutive trials were of the same condition.

Each trial started with the presentation of a central fixation cross of variable length between 200 and 1500 ms followed by the picture with the fish until a response was given but maximally for four seconds (time-out). In addition to the stimulus pictures we created animations as feedback: the target fish would move to the side of the pressed button accompanied by a fanfare (after any flanking fish had disappeared) upon a correct response. For incorrect responses or in the case of a missing response, all flanking fish disappeared but the target fish remained in its position along with a bubbling sound. Both feedback animations had a length of 1200 ms.

At the beginning of the experiment an initial display showed several fish of different colors in an aquarium. The experimenter told the child a story about how hungry the fish are and explained that the game would be to help the fish get to the food. Then the experimenter introduced the task by going step-by-step through one demonstration trial (of the neutral condition). In a first practice block, six items served to illustrate which fish was the target fish (the one appearing behind the fixation cross), which button had to be pressed, and what happened when the correct or incorrect button was pressed. Here, children had unlimited time to respond. The second practice block with another six items was used to encourage children to respond quickly within the four seconds. The items in this block were repeated until a correct answer was given. Before the start of the eight test blocks the children were shown eight contours in the form of a fish (one for each block). They were told that for each completed block one contour would be replaced by a colorful fish and that after all the contours were filled the game would be over. This screen appeared after each block with an additional applause sound after the final block. During the test, each trial started immediately after the feedback animation from the previous one.

The digit span was administered in a computerized version as well to ensure comparable presentation conditions for all participants. The presentation of the pre-recorded digits was delivered by the DMDX software, which was operated by the experimenter. The digits were pre-recorded one by one by a male native speaker of German in a neutral voice. We compiled sequences from the single recordings with a rate of one digit per second. Within a sequence a digit was never repeated. All digits were one syllable long (to avoid pronunciation duration effects, cf. Chen and Stevenson [50]). The digit span test was introduced to the child as the “parrot game,” in which the child takes the role of a parrot and repeats everything. The test started with two practice trials with a sequence length of two. If the child understood the task and was able to repeat two out of four practice trials correctly, the test began with a sequence length of two. For each sequence length, there were three sequences. If two sequences of a given length were repeated correctly, the experimenter increased the sequence length by one. If there was more than one error (in the repeated digits or in the order) for a given length, the test was terminated and children saw a picture of a parrot and heard applause. Repetitions were not allowed in the test trials. The dependent variable used for analysis was the digit span, i.e. the sequence length for which a child repeated two sequences correctly.

### Results

#### Sentence-picture verification

**Fig 6:**
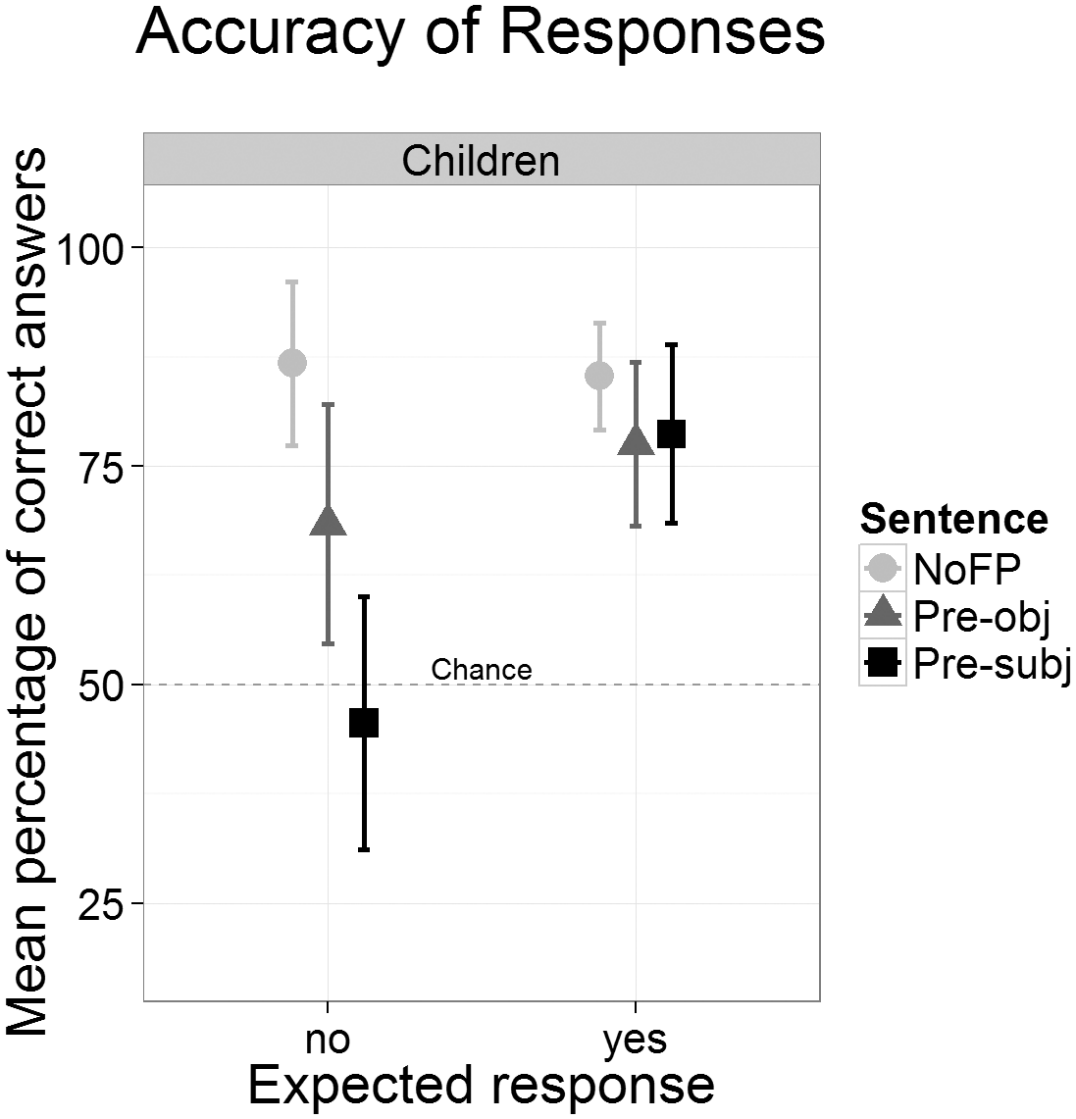
Mean accuracy scores for each sentence type and expected response. Error bars denote two standard errors.

Fig 6 displays the mean response accuracies. For each condition (sentence type by expected answer) a child could give up to eight responses. Children gave significantly more correct *yes* than *no* responses (80.5% vs. 66.9%: *t*(33) = 2.19, *p* < .05). The accuracy scores did not differ between the sentence types for expected *yes* responses (FP: 85.3%, Pre-obj: 77.6%, Pre-subj: 78.7%, all *t*<1.62, all *p*>.115). There is a graded performance for the expected *no* responses. Accuracy scores were higher for NoFP compared to Pre-obj (86.8% vs. 68.4%, *t* = 3.13, *p* < .01) and also higher for Pre-obj compared to Pre-subj sentences (68.4% vs. 45.6%, *t* = 4.17, *p* < .001). Only the group performance for Pre-subj sentences did not differ from the chance level (*t*(33)<1, *p* = .546). When classifying children as passing the FP sentences (at least 75% correct responses), there were 22 Pre-obj passers and 14 Pre-subj passers (of the 12 Pre-obj non-passers, 5 never gave a correct response; among the 20 Pre-subj non-passers, there were 12 without a single correct response).

#### Eye gaze data

**Fig 7:**
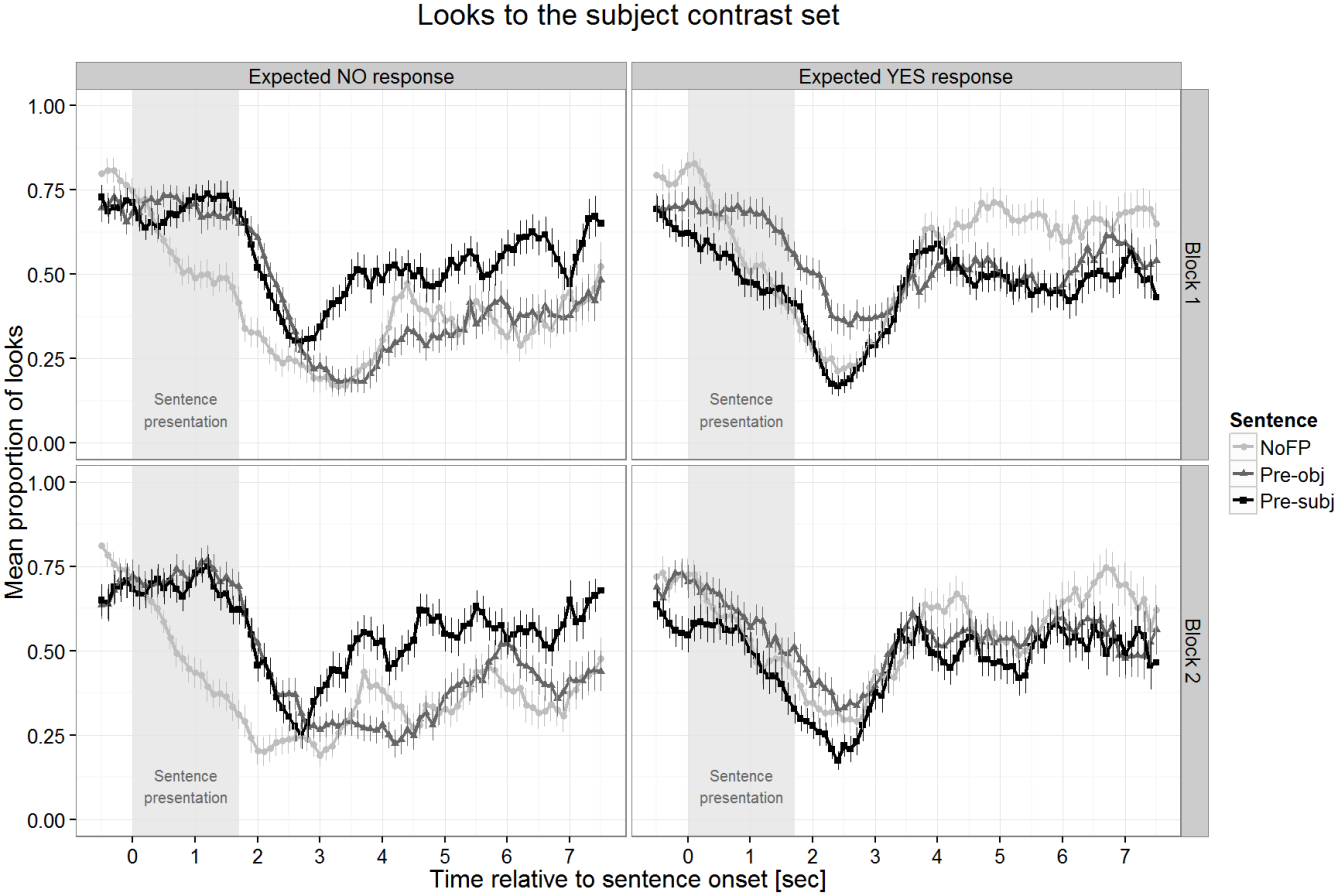
Looks to the subject alternative set plotted separately for block and expected response.

Data analysis proceeded exactly as in Study 1 using the same AoIs and removing invalid eye tracking data (about 9% of all data). After applying the criterion to each trial (less than 50% track loss), on average 46.4 trials (out of 48) remained in the analysis sample. The data from one girl were removed completely because fewer than half of all trials survived the 50% criterion. Fig 7 plots the time course of these looking proportions relative to sentence onset for all trials.

At sentence onset the looking proportions to the subject alternative set were at roughly .75, a value predicted by chance. Looks to these non-mentioned characters decreased after presentation of the test sentence containing the FP *only* (Pre-subj and Pre-obj) and even earlier in NoFP sentences. About 3 seconds after sentence onset looking proportions in Pre-obj sentences started to diverge from the ones in Pre-subj sentences for *no* response trials. We assessed the differences in looking proportions in analogy to Study 1 by fitting a linear mixed-effects model for each of the one-second windows separately. Apart from the absence of the predictor Age, the model specification is identical to Study 1: three within-participant factors for sentence type, block, and expected response in which Pre-subj is coded as the base of a treatment contrast to be able to compare this condition to both NoFP and Pre-obj sentences. block was included to follow up the block effect found in Study 1. In Study 2 this factor just represented the first vs. the second half of the experiment without a change in task. The values of the looking proportions are given in Table 2.

**Table 2:** Mean proportions of looks to the subject alternative set.

Sentence	0	1	2	3	4	5	6	7
Pre-subj	.627	.547	.295	.463	.537	.508	.526	.556
Pre-obj	.693	.625	.384	.369***	.427***	.469***	.475**	.509**
NoFP	.638	.419***	.254*	.352***	.509***	.514***	.499***	.557

The proportions are aggregated over both blocks and both expected responses for each time window after the onset of the sentence. A window has a duration of one second, i.e. window 0 starts with the onset of the test sentence and comprises the first second after sentence onset. Significant differences to the Pre-subj sentences are marked by asterisks:

*: *p* < .05,

**: *p* < .01,

***: *p* < .001.

**Fig 8:**
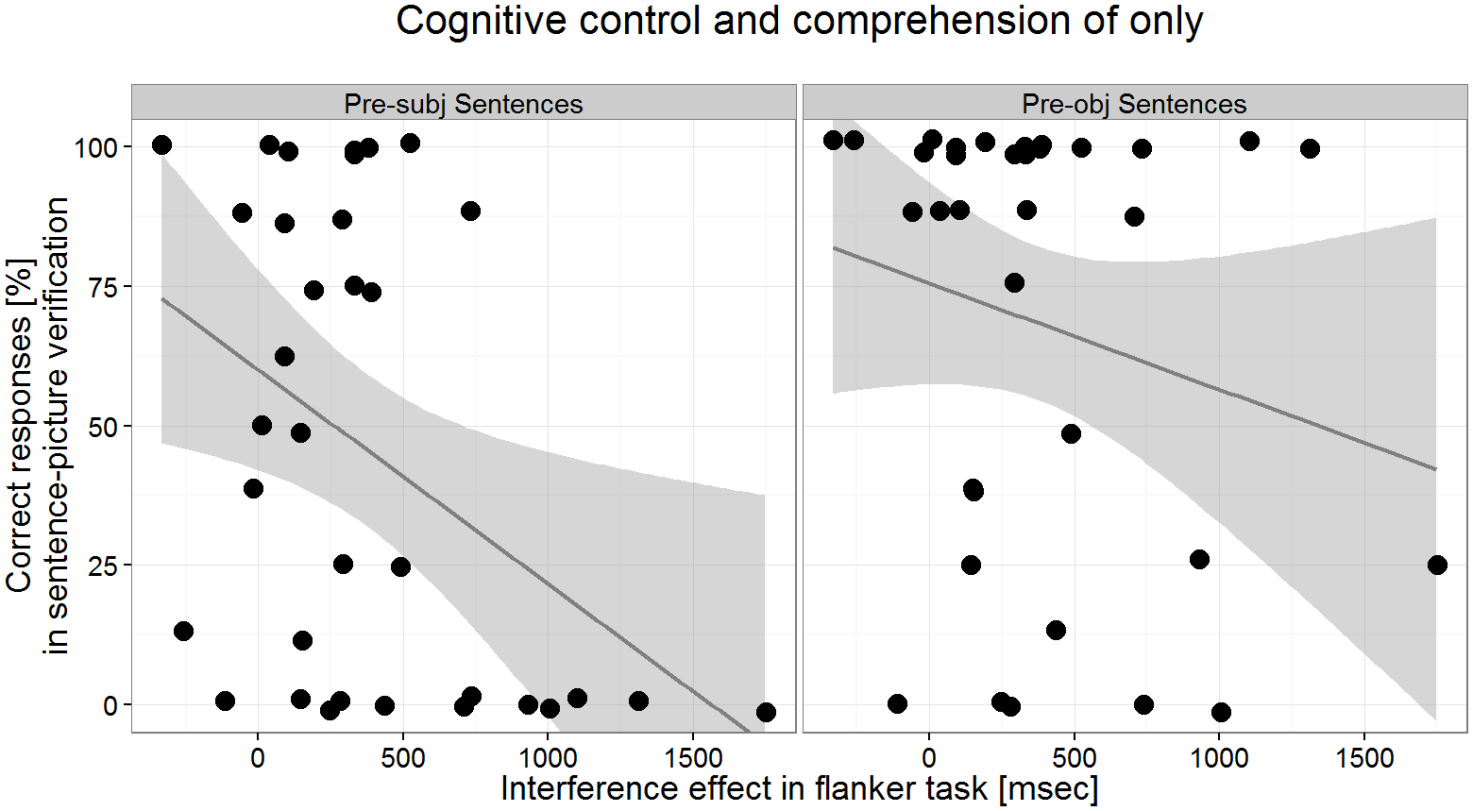
Relation between cognitive control and the comprehension of ‘only’. The interference effect as a measure of cognitive control is plotted against the accuracy in the sentence-picture verification for the non-matching sentences containing *only* separated by position (pre-subject sentences on the left, pre-object sentences on the right).

We report only significant effects for Sentence Type and interactions with this factor (the complete model parameters for the fixed effects can be found in the table in S2 Table), which were present in all windows except window 0. Looking proportions to the subject alternative set were higher in Pre-subj compared to Pre-obj sentences for all windows from window 3 onwards (all *t*>3.22, all *p* < .01). This effect was modulated by expected response (Fig 8) such that in trials with expected *yes* responses this difference disappeared in all these windows (all *t*>3.90, all *p* < .001). The comparison between Pre-subj vs. NoFP revealed that for windows 1 to 7 looking proportions to the subject alternative set were significantly higher after the presentation of Pre-subj sentences (all *t*>2.7, all *p* < .05). This difference was influenced by expected response in the same way as in the previous comparison as the *yes* response trials did not show this pattern (all *t*>2.55, all *p* < .05). block did not interact with sentence type at all.

#### Flanker task

Missing reactions (latencies longer than 4000 ms) were counted as incorrect, which affected 4% of the data. Response times below 300 ms were considered to be too short to be a response to the stimulus and were therefore removed. This affected 0.6% of the data. Only correct responses were included in the analysis of the reaction times. Accuracy in all three conditions was above chance (neutral: 91%; *t*(33) = 25.5, *p* < .001; congruent: 91%; *t*(33) = 24.1, *p* < .001; incongruent: 71%; *t*(33) = 4.58, *p* < .001). While the accuracy scores in neutral and congruent items were not different from each other (*t*(33) = 1.32, *p* = .197), they were lower for incongruent compared to congruent items (*t*(33) = 4.38, *p* < .001). The response times differed across conditions for both comparisons (neutral: 1284 ms, SD = 326); congruent: 1466 ms, SD = 403; incongruent: 1842 ms, SD = 525). As expected, we found the two effects: the flanker effect (congruent vs. neutral: *t*(33) = 4.29, *p* < .001) shows that additional fish even without conflicting information slowed down the response. The interference effect (incongruent trials with slower reaction times than congruent ones, *t*(33) = 4.90, *p* < .001) shows that conflicting information slowed down the response even more.

The results of the flanker task indicate that the task was suited to 4-year-olds as they performed above chance in all conditions. In the next analysis step we tested if the individual interference effects (mean: 376 ms, range: –332 to 1750) are related to the ability to correctly interpret sentences with *only*.

#### Digit span

For two of the 34 children we were unable to obtain the digit span: one child refused to repeat any numbers and another did not understand the instructions. The digit span was measured as the maximum length of a sequence for which there were two correct sequence repetitions. This value ranged between 2 and 4 with a mean of 2.8 (SD = 0.7).

#### The relation between sentence-picture verification and cognitive measures

We fitted a linear mixed-effects model to estimate the influence of cognitive control on the responses in the sentence-picture verification task. An advantage over regular correlations is that we can simultaneously control for age effects and respect the data structure of the sentence-picture verification. The dependent variable was the accuracy of the response (correct or incorrect) in the non-matching sentences containing *only*. In addition to the factor position of *only* (Pre-subj and Pre-obj) and the cognitive control measure (interference effect in seconds), we included age (in months) and digit span as estimates. The model (complete model specification and list of fixed effects parameters are given in the table in S3 Table) confirmed the decrease of about 24% in response accuracy in Pre-subj compared to Pre-obj sentences (*z* = 6.81, *p* < .001). Cognitive control also affected accuracy but differently for Pre-subj and Pre-object sentences. While a larger interference effect (a slowdown of 1 second) reduced accuracy in Pre-subj sentences significantly by about 35% (*z* = 2.80, *p* < .01), the reduction of about 15% for Pre-obj sentences was not significantly different from zero (*z*<1). Fig 8 illustrates the relation between the performance in the sentence-picture verification task and the flanker task for each individual child.

The forward digit span affected accuracy in both Pre-obj and Pre-subj sentences in the same way as indicated by a non-significant interaction (*z*<1): an increase in digit span by one increased accuracy by about 30% (*z* = 4.14, *p* < .001). Age had no significant effect on the accuracy scores (all *z*<1.52).

### Discussion of Study 2

The results from the eye tracking and the sentence-picture verification task of Study 2 replicate those of the children from Study 1: while the eye gaze data show an enhanced proportion of looks to the subject alternative set after listening to a pre-subject *only* sentence, the group data from the sentence-verification task do not evidence a correct interpretation of these sentences. Having tested different children in the two studies, we are confident in concluding that first, our finding that the FP position in the test sentences guided the visual attention of the children to the display is reliable, and second, that the divergence between the eye gaze data and the performance in the sentence-picture verification task is a robust pattern. In Study 2 there was no difference between both testing blocks in contrast to Study 1. This suggests that the unexpected block effect in Study 1 was neither an order effect nor a consequence of the judgment task because this task was present for both blocks in Study 2. Rather, it seems to have been the change in procedure (including a new task mid-experiment) that led to different looking patterns in the second block of Study 1. The differential looking patterns for expected *yes* and *no* responses occurred in both studies. We hypothesize that this difference occurred because the introduction of the alternative set may have led participants to anticipate the upcoming test sentence. As a result, the need to re-check the visual information became moot for anticipated sentences as in the case of expected *yes* responses. If this explanation is on the right track then removing the introduction of the alternative set should make the looking patterns for the expected *yes* and the expected *no* response trials more similar to each other.

The main difference with regard to the verification part of the two studies lies in the overall higher performance that children showed in the *no* response condition in Study 2 compared to Study 1. We assume that this difference is due to the fact that children had to provide a response over the whole experiment in Study 2 while this was only the case for the second half of Study 1. Only passively listening to the sentences during the first half of the experiment in Study 1 might have had a detrimental effect on children’s general attention.

Concerning the primary aim of Study 2, our results show that the individual performance in the flanker task is related to the accuracy of rejecting a sentence with Pre-subject *only*. Consistent with our hypothesis, we found that less cognitive control (a larger slowdown in response time with conflicting information in the flanker task) is correlated with lower accuracy scores in pre-subject but not in pre-object *only* sentences. Adding to the findings by Minai et al. [40], our results thus suggest that inhibitory control as a rather late developing general cognitive ability may have a substantial impact on the outcome of tasks that are mainly considered to measure children’s linguistic abilities. At this point, direction and causality of this relation remains unclear and should be clarified by further studies. However, we propose that this relationship is not a mere general effect of cognitive development. For one, we did not find an effect of age on the ability to verify the sentences containing *only*. Furthermore, the presence of a working memory effect on overall accuracy scores (i.e. both for pre-subject and pre-object *only* sentences) shows that this measure seems to pick up general cognitive abilities that are relevant to solving the verification task. As described above, working memory was not differentially associated to the children’s performance level for the two sentence types. This suggests that the two sentence types do not differ with respect to their working memory requirements and that holding the linguistic representation in memory while evaluating it against the visual information may not be the crucial cognitive factor that is related to better performance for the pre-object compared to the pre-subject sentences in the verification task. Instead, our findings point to the relevance of cognitive control (especially inhibitory control) in the process of deciding whether a pre-subject *only* sentence matches a visual scenario that would adequately be described by a corresponding pre-object *only* sentence. We consider the pre-subject *only* sentence as non-canonical because the focused element appears in a syntactic position that violates the information structure default (subject = topic, object = focus). It is these non-canonical linguistic representations which require inhibitory control abilities for interpretation since competition with canonical (i.e. dominant) representations arise that need to be suppressed. We will come back to this in the General Discussion.

## General Discussion

Summarizing the main results of our studies we found that German-learning 4-year-olds’ attention is guided by the occurrence and the syntactic position of the focus particle *only* in a visual world paradigm. The looking patterns of adults and children bear a remarkable similarity. However, overt decisions about the match between the linguistic and visual information did not show the correct integration of the focus particle into the sentence interpretation, especially in the case of subject associated *only*. Moreover, children’s ability to reject these sentences in the case of a mismatch was related to a specific component of cognitive control, i.e. the inhibition mechanism. In the following we will first discuss the implications of our findings for research on children’s acquisition of this FP as outlined in the introduction. Second, we will discuss implications for research on language acquisition from a more general perspective.

Paterson and colleagues [11, 14] argue that immature pragmatic abilities hinder children in establishing and mentally representing alternative sets when such a set is not available from prior discourse context but has to be inferred from other sources. In our experiments, the relevant alternative sets were verbally introduced prior to the presentation of the test sentences. Nevertheless, the children showed non-adult-like performance levels in the sentence-verification task. As we did not run a control experiment without this verbal introduction, we cannot be sure that our procedure made the alternative set salient enough to overcome the problems caused by an immatureness of pragmatic abilities as proposed by Paterson et al. But most importantly, we found that under the same experimental conditions the eye tracking data revealed that children take the subject alternative set into account when evaluating a sentence with pre-subject *only*, but that this does not necessarily enter the final interpretation that children assign to the sentence. Thus, our response data from the sentence-picture verification task could in principle still be interpreted in line with Paterson et al.’s account, but again our data show an uneven performance pattern depending on whether the alternative set has to be established for the subject or the object of the sentence–an asymmetry that is not predicted by this proposal.

With respect to the hypothesis that children’s problems with sentences containing *only* are caused by problems with associating the particle to the correct constituent as signaled by the sentence structure, the picture is slightly more complicated. Our results from the sentence-picture verification task replicate previous findings of an asymmetric performance for subject- and object-associated *only*. In both of our studies children gave more correct *no* responses to pre-object than to pre-subject *only* sentences. As presented in the introduction, Notley and colleagues [9] as well as Zhou and Crain [10] have argued that this asymmetry reflects children’s still non-adult-like syntactic system, which allows the pre-subject *only* to take scope over the VP/object. Yet our eye tracking data reveal a systematic effect of the position of the focus particle on children’s gaze to the corresponding visually presented alternative set, i.e. to the subject alternative set when presented with a pre-subject *only* sentence. This conflicts with the assumption that children’s syntactic knowledge is not yet sufficiently developed to obey the scope restrictions that are connected to the pre-subject position of the FP.

Overall, we consider our data to be most compatible with Müller et al.’s [17] assumption according to which children’s lower performance in verifying pre-subject *only* sentences is related to the mismatch between the sentence structure and the default assignment of focus to the sentence object. Our current data allow us to formulate this proposal–which was only based on children’s response performance in a sentence-picture verification task–in a more specific way. We assume that children initially parse the sentence structure correctly as suggested by the eye tracking data and build a representation of the sentence meaning that includes the contribution of the focus particle and its structural position, thereby associating it to the corresponding sentence constituent. But as we have already argued above, this interpretation competes with the pragmatically driven bias to treat the sentence object as the focus to which the particle has to be associated. This conflict needs to be resolved when deciding whether the sentence matches the information provided by the picture. Whether this competition between different interpretations leads to an ignoring of the focus particle–as Müller et al. [17] assume–or whether the children go for the default object association–as is argued by Notley et al. [9] and Zhou and Crain [10]–cannot be decided based on our data as both would lead to an acceptance of the false sentences.

That children go for a default interpretation in the case of potentially competing interpretations is also reported by Zhou et al. [31]. Remember that in their results children’s sentence-picture judgments consistently reflected a focus assignment to the modifier while in the same task the adults switched between modifier and head noun assignment based on the placement of the focus accent. As the modifier is the most deeply embedded part in the modifier-head construction, stress/focus assignment to the modifier is in fact the default reading, which seemed to override the prosodic information for the children. Considering the modifier assignment as the default was further supported by the observation that adults clearly favored this reading when presented with the sentences in written form (i.e. without the disambiguating prosodic information). However, comparable to our study, children’s eye gazes indicated an adult-like interpretation of the sentences. Thus, it should be noted again that we consider children’s “misinterpretations” in judgments as a task-induced effect in which a more frequent and thus strongly competing default competitor “wins” over a weaker non-canonical interpretation.

In light of this assumption, children’s varying performance in non-canonical structures with non-default interpretations can also be explained, as different sources of information may strengthen the weaker candidate. Zhou and Crain’s [10] findings of better achievements in pre-subject *only* sentences with negation suggest that the negation provides children with a strong syntactic cue that favors the correct reading over a competing default. The same holds for contextual effects: if the favored default reading is not compatible with the previous discourse context or the goal of the discourse, its competitive effect on the correct reading may be reduced and thus better performance is observed [15].

Even though our studies on children’s comprehension of sentences with the FP *only* did not test ambiguous structures, the relation to the outcomes of the flanker task as a measurement of inhibitory control supports our interpretation. If the default interpretation competes with the structurally induced reading of the sentence, the default interpretation must be inhibited to arrive at a correct decision in the sentence-picture verification task. As we have shown, better developed inhibitory skills were specifically related to children’s ability to correctly reject the pre-subject *only* sentences in a scenario in which a pre-object sentence would be true, i.e. in a scenario that requires the inhibition of a strong competitor–which is additionally strengthened by the information on the visual display. Importantly, children’s performance with the pre-object *only* sentences was not related to their inhibitory skills. As in this case the reading of the presented sentence corresponds to the default reading, no competition between different readings and consequently no requirement for inhibitory skills arise.

To provide further evidence for our approach, it would be interesting to investigate which interpretation children would assign to pre-subject *only* sentences in which the FP association is ambiguous within the subject constituent. Similarly to Zhou et al. [31], testing German sentences with a subject modifier like *Only the mouse with the hat is dancing* would be informative. In this structure, the FP has scope over the head noun (*the mouse*) as well as over the modifier (*hat*). Based on the principle that the most embedded constituent is the default focus position that carries the focus accent, the default interpretation would associate the FP to the whole constituent or to the modifier. However, a shift of the focus accent to the head noun restricts the association of the FP to the head. According to our proposal, children should perform well with the sentences in which the focus accent is placed on the modifier. But fewer correct answers and a correlation of the performance with inhibitory skills would be predicted for sentences in which the focus accent signals an association of the FP to the head noun.

To conclude our discussion on children’s comprehension of sentences with pre-subject *only*, we interpret our findings in the following way: by the age of 4 years, German-learning children have the linguistic (syntactic and pragmatic) competence for a correct interpretation of sentences involving the FP *only* in either a pre-subject or pre-object position. For the first time, our study has provided evidence that children’s low performance in tasks that require an overt decision about the match of a sentence involving an FP to a non-verbal scenario does not necessarily reflect this competence because still developing general cognitive processes such as cognitive control may mask these abilities.

This may have implications for language acquisition research in general. So far research on language acquisition, especially on higher order linguistic capacities involving syntax, semantics, and pragmatics, has considered language development in a rather modular view without taking the ongoing development in other cognitive, non-linguistic areas into account. This is especially problematic if methods are employed that require additional extra-linguistic skills, which are known to develop rather late during early childhood as we have shown for the sentence-picture verification task. In research on children’s language comprehension, tasks like these, including truth value judgment or picture-selection, which all may be affected in a similar way by general cognitive abilities, are widely applied. Our finding suggests that a closer evaluation of the demands that these tasks place on the different cognitive systems in the developing child and the contribution of the involved systems to children’s performance patterns is essential for a better understanding of language development.

## Supporting Information

S1 Table Model output for the fixed effects in Study 1. (DOCX)

S2 Table Model output for the fixed effects in Study 2. (DOCX)

S3 Table Model output for the fixed effects of the influence of the flanker task on the sentence-picture verfication in Study 2. (DOCX)

S4 Table Complete sentence material used in Study 1 and Study 2. (DOCX)
