# Supplementary material for: Children’s Comprehension of Sentences with Focus Particles and the Role of Cognitive Control: An Eye Tracking Study with German-Learning 4-Year-Olds
Source: PLoS One. 2016 Mar 1;11(3):e0149870. doi: 10.1371/journal.pone.0149870 (PMC4773164; doi:10.1371/journal.pone.0149870)
Supplement: S1 Table — (DOCX) [file pone.0149870.s001.docx]

**S1 Table. Model output for the fixed effects in Study 1.**

| Window | Predictor | Estimate | SE | df | t-value | p-value |
| --- | --- | --- | --- | --- | --- | --- |
| 0 | Intercept | .700 | .038 | 104.4 | 18.209 | <.001 |
| 0 | NoFP | -.045 | .052 | 56.4 | -0.874 | .386 |
| 0 | Pre-obj | -.024 | .048 | 89.6 | -0.503 | .617 |
| 0 | Block | -.040 | .027 | 1476 | -1.525 | .127 |
| 0 | Exp.Response | -.060 | .047 | 88.8 | -1.256 | .213 |
| 0 | Adults | -.070 | .046 | 155.9 | -1.539 | .126 |
| 0 | NoFP:Block | .084 | .038 | 1472 | 2.241 | .025 |
| 0 | Pre-obj:Block | .067 | .038 | 1472 | 1.778 | .076 |
| 0 | NoFP:Exp.Response | .037 | .073 | 56.3 | 0.51 | .612 |
| 0 | Pre-obj:Exp.Response | .085 | .067 | 88.9 | 1.262 | .210 |
| 0 | Block:Exp.Response | -.003 | .037 | 1473 | -0.074 | .941 |
| 0 | NoFP:Adults | .070 | .053 | 1471 | 1.322 | .186 |
| 0 | Pre-obj:Adults | .086 | .053 | 1472 | 1.639 | .101 |
| 0 | Block:Adults | .054 | .037 | 1476 | 1.451 | .147 |
| 0 | Exp.Response:Adults | .136 | .053 | 1471 | 2.582 | .010 |
| 0 | NoFP:Block:Exp.Response | .006 | .053 | 1472 | 0.121 | .903 |
| 0 | Pre-obj:Block:Exp.Response | -.009 | .053 | 1473 | -0.172 | .863 |
| 0 | NoFP:Block:Adults | -.017 | .053 | 1472 | -0.325 | .745 |
| 0 | Pre-obj:Block:Adults | -.014 | .053 | 1472 | -0.257 | .797 |
| 0 | NoFP:Exp.Response:Adults | -.041 | .074 | 1471 | -0.544 | .586 |
| 0 | Pre-obj:Exp.Response:Adults | -.146 | .074 | 1471 | -1.964 | .050 |
| 0 | Block:Exp.Response:Adults | .024 | .053 | 1472 | 0.451 | .652 |
| 0 | NoFP:Block:Exp.Response:Adults | -.081 | .074 | 1472 | -1.093 | .275 |
| 0 | Pre-obj:Block:Exp.Response:Adults | -.055 | .074 | 1473 | -0.742 | .458 |
| 1 | Intercept | .654 | .041 | 103.1 | 15.964 | <.001 |
| 1 | NoFP | -.265 | .055 | 56.6 | -4.797 | <.001 |
| 1 | Pre-obj | .027 | .051 | 92.3 | 0.523 | .602 |
| 1 | Block | -.044 | .029 | 1471 | -1.502 | .133 |
| 1 | Exp.Response | -.187 | .051 | 91 | -3.68 | <.001 |
| 1 | Adults | -.184 | .049 | 160.5 | -3.719 | <.001 |
| 1 | NoFP:Block | .086 | .041 | 1468 | 2.07 | .039 |
| 1 | Pre-obj:Block | .080 | .041 | 1468 | 1.942 | .052 |
| 1 | NoFP:Exp.Response | .168 | .078 | 56.5 | 2.15 | .036 |
| 1 | Pre-obj:Exp.Response | .039 | .072 | 91.6 | 0.544 | .588 |
| 1 | Block:Exp.Response | .062 | .041 | 1469 | 1.503 | .133 |
| 1 | NoFP:Adults | .048 | .058 | 1467 | 0.826 | .409 |
| 1 | Pre-obj:Adults | -.049 | .058 | 1468 | -0.853 | .394 |
| 1 | Block:Adults | .076 | .041 | 1471 | 1.869 | .062 |
| 1 | Exp.Response:Adults | .140 | .058 | 1467 | 2.418 | .016 |
| 1 | NoFP:Block:Exp.Response | .006 | .058 | 1468 | 0.102 | .919 |
| 1 | Pre-obj:Block:Exp.Response | -.101 | .058 | 1469 | -1.727 | .084 |
| 1 | NoFP:Block:Adults | -.058 | .058 | 1467 | -1.002 | .317 |
| 1 | Pre-obj:Block:Adults | .015 | .058 | 1468 | 0.253 | .800 |
| 1 | NoFP:Exp.Response:Adults | -.064 | .082 | 1467 | -0.783 | .434 |
| 1 | Pre-obj:Exp.Response:Adults | .019 | .082 | 1467 | 0.235 | .814 |
| 1 | Block:Exp.Response:Adults | -.031 | .058 | 1468 | -0.545 | .586 |
| 1 | NoFP:Block:Exp.Response:Adults | -.027 | .082 | 1467 | -0.326 | .745 |
| 1 | Pre-obj:Block:Exp.Response:Adults | -.020 | .082 | 1468 | -0.244 | .808 |
| 2 | Intercept | .368 | .035 | 106.1 | 10.396 | <.001 |
| 2 | NoFP | -.118 | .045 | 58.7 | -2.648 | .010 |
| 2 | Pre-obj | .065 | .042 | 104.2 | 1.548 | .125 |
| 2 | Block | -.044 | .026 | 1472 | -1.674 | .094 |
| 2 | Exp.Response | -.121 | .042 | 103.8 | -2.889 | .005 |
| 2 | Adults | -.149 | .046 | 143.6 | -3.26 | .001 |
| 2 | NoFP:Block | .049 | .037 | 1470 | 1.312 | .190 |
| 2 | Pre-obj:Block | .054 | .037 | 1470 | 1.47 | .142 |
| 2 | NoFP:Exp.Response | .156 | .063 | 58.6 | 2.487 | .016 |
| 2 | Pre-obj:Exp.Response | .072 | .059 | 104 | 1.218 | .226 |
| 2 | Block:Exp.Response | -.024 | .037 | 1471 | -0.663 | .508 |
| 2 | NoFP:Adults | -.063 | .052 | 1469 | -1.208 | .227 |
| 2 | Pre-obj:Adults | -.136 | .052 | 1469 | -2.618 | .009 |
| 2 | Block:Adults | .009 | .037 | 1473 | 0.251 | .802 |
| 2 | Exp.Response:Adults | .081 | .052 | 1469 | 1.565 | .118 |
| 2 | NoFP:Block:Exp.Response | .003 | .052 | 1470 | 0.056 | .955 |
| 2 | Pre-obj:Block:Exp.Response | -.050 | .052 | 1471 | -0.96 | .337 |
| 2 | NoFP:Block:Adults | .000 | .052 | 1469 | 0.001 | .999 |
| 2 | Pre-obj:Block:Adults | .019 | .052 | 1470 | 0.359 | .720 |
| 2 | NoFP:Exp.Response:Adults | -.067 | .073 | 1469 | -0.91 | .363 |
| 2 | Pre-obj:Exp.Response:Adults | .069 | .073 | 1469 | 0.946 | .345 |
| 2 | Block:Exp.Response:Adults | .040 | .052 | 1470 | 0.765 | .444 |
| 2 | NoFP:Block:Exp.Response:Adults | -.014 | .073 | 1469 | -0.19 | .849 |
| 2 | Pre-obj:Block:Exp.Response:Adults | .018 | .073 | 1471 | 0.242 | .809 |
| 3 | Intercept | .457 | .040 | 100.3 | 11.46 | <.001 |
| 3 | NoFP | -.184 | .053 | 53 | -3.45 | .001 |
| 3 | Pre-obj | -.120 | .049 | 86.2 | -2.437 | .017 |
| 3 | Block | .039 | .029 | 1466 | 1.354 | .176 |
| 3 | Exp.Response | .042 | .049 | 85.8 | 0.852 | .397 |
| 3 | Adults | -.153 | .049 | 163.3 | -3.131 | .002 |
| 3 | NoFP:Block | -.027 | .041 | 1464 | -0.654 | .513 |
| 3 | Pre-obj:Block | -.049 | .040 | 1464 | -1.217 | .224 |
| 3 | NoFP:Exp.Response | .192 | .075 | 53.1 | 2.539 | .014 |
| 3 | Pre-obj:Exp.Response | .045 | .070 | 86.2 | 0.652 | .516 |
| 3 | Block:Exp.Response | -.086 | .040 | 1464 | -2.139 | .033 |
| 3 | NoFP:Adults | .026 | .057 | 1463 | 0.452 | .651 |
| 3 | Pre-obj:Adults | -.010 | .057 | 1463 | -0.181 | .856 |
| 3 | Block:Adults | -.007 | .040 | 1467 | -0.177 | .859 |
| 3 | Exp.Response:Adults | -.027 | .057 | 1463 | -0.477 | .633 |
| 3 | NoFP:Block:Exp.Response | .019 | .058 | 1464 | 0.332 | .740 |
| 3 | Pre-obj:Block:Exp.Response | .017 | .057 | 1465 | 0.291 | .771 |
| 3 | NoFP:Block:Adults | -.026 | .057 | 1464 | -0.458 | .647 |
| 3 | Pre-obj:Block:Adults | .019 | .057 | 1464 | 0.336 | .737 |
| 3 | NoFP:Exp.Response:Adults | -.129 | .081 | 1463 | -1.598 | .110 |
| 3 | Pre-obj:Exp.Response:Adults | .031 | .080 | 1463 | 0.384 | .701 |
| 3 | Block:Exp.Response:Adults | -.016 | .057 | 1464 | -0.273 | .785 |
| 3 | NoFP:Block:Exp.Response:Adults | .060 | .081 | 1463 | 0.748 | .454 |
| 3 | Pre-obj:Block:Exp.Response:Adults | .062 | .080 | 1464 | 0.766 | .444 |
| 4 | Intercept | .637 | .040 | 115.9 | 15.818 | <.001 |
| 4 | NoFP | -.221 | .051 | 69.5 | -4.321 | <.001 |
| 4 | Pre-obj | -.230 | .048 | 122.9 | -4.745 | <.001 |
| 4 | Block | .044 | .031 | 1468.1 | 1.455 | .146 |
| 4 | Exp.Response | -.018 | .048 | 122.2 | -0.37 | .712 |
| 4 | Adults | -.208 | .053 | 156.9 | -3.97 | <.001 |
| 4 | NoFP:Block | -.113 | .044 | 1465.4 | -2.589 | .010 |
| 4 | Pre-obj:Block | -.114 | .043 | 1466.2 | -2.622 | .009 |
| 4 | NoFP:Exp.Response | .340 | .072 | 68.7 | 4.712 | <.001 |
| 4 | Pre-obj:Exp.Response | .260 | .068 | 122.5 | 3.801 | <.001 |
| 4 | Block:Exp.Response | -.027 | .043 | 1466.6 | -0.615 | .539 |
| 4 | NoFP:Adults | -.012 | .061 | 1465.3 | -0.195 | .845 |
| 4 | Pre-obj:Adults | .027 | .061 | 1465.1 | 0.445 | .657 |
| 4 | Block:Adults | .021 | .043 | 1468.7 | 0.485 | .628 |
| 4 | Exp.Response:Adults | -.168 | .061 | 1464.8 | -2.761 | .006 |
| 4 | NoFP:Block:Exp.Response | .084 | .062 | 1465.7 | 1.355 | .176 |
| 4 | Pre-obj:Block:Exp.Response | .048 | .061 | 1467 | 0.789 | .430 |
| 4 | NoFP:Block:Adults | .046 | .061 | 1464.9 | 0.758 | .449 |
| 4 | Pre-obj:Block:Adults | .037 | .061 | 1465.8 | 0.604 | .546 |
| 4 | NoFP:Exp.Response:Adults | -.019 | .086 | 1465 | -0.226 | .821 |
| 4 | Pre-obj:Exp.Response:Adults | .019 | .086 | 1464.9 | 0.22 | .826 |
| 4 | Block:Exp.Response:Adults | -.053 | .061 | 1466 | -0.877 | .381 |
| 4 | NoFP:Block:Exp.Response:Adults | -.015 | .086 | 1465 | -0.17 | .865 |
| 4 | Pre-obj:Block:Exp.Response:Adults | .052 | .086 | 1466.4 | 0.611 | .541 |
| 5 | Intercept | .646 | .041 | 110.4 | 15.771 | <.001 |
| 5 | NoFP | -.243 | .051 | 61 | -4.726 | <.001 |
| 5 | Pre-obj | -.136 | .049 | 112 | -2.776 | .006 |
| 5 | Block | .037 | .032 | 1460 | 1.163 | .245 |
| 5 | Exp.Response | -.031 | .049 | 112.2 | -0.637 | .525 |
| 5 | Adults | -.251 | .054 | 165.8 | -4.624 | <.001 |
| 5 | NoFP:Block | -.063 | .046 | 1461 | -1.37 | .171 |
| 5 | Pre-obj:Block | -.023 | .045 | 1457 | -0.498 | .618 |
| 5 | NoFP:Exp.Response | .363 | .073 | 60.5 | 4.998 | <.001 |
| 5 | Pre-obj:Exp.Response | .164 | .070 | 112.4 | 2.356 | .020 |
| 5 | Block:Exp.Response | -.045 | .045 | 1458 | -0.996 | .319 |
| 5 | NoFP:Adults | .096 | .064 | 1455 | 1.511 | .131 |
| 5 | Pre-obj:Adults | .044 | .063 | 1455 | 0.694 | .488 |
| 5 | Block:Adults | -.033 | .045 | 1460 | -0.727 | .467 |
| 5 | Exp.Response:Adults | -.050 | .063 | 1455 | -0.795 | .427 |
| 5 | NoFP:Block:Exp.Response | .121 | .064 | 1459 | 1.879 | .060 |
| 5 | Pre-obj:Block:Exp.Response | .048 | .064 | 1458 | 0.747 | .455 |
| 5 | NoFP:Block:Adults | .033 | .064 | 1458 | 0.518 | .604 |
| 5 | Pre-obj:Block:Adults | .019 | .063 | 1457 | 0.306 | .760 |
| 5 | NoFP:Exp.Response:Adults | -.160 | .090 | 1455 | -1.779 | .076 |
| 5 | Pre-obj:Exp.Response:Adults | -.079 | .089 | 1455 | -0.884 | .377 |
| 5 | Block:Exp.Response:Adults | .013 | .063 | 1457 | 0.21 | .834 |
| 5 | NoFP:Block:Exp.Response:Adults | -.075 | .090 | 1457 | -0.834 | .405 |
| 5 | Pre-obj:Block:Exp.Response:Adults | .010 | .089 | 1457 | 0.108 | .914 |
| 6 | Intercept | .662 | .039 | 123 | 16.77 | <.001 |
| 6 | NoFP | -.217 | .049 | 78.7 | -4.467 | <.001 |
| 6 | Pre-obj | -.081 | .047 | 153.6 | -1.702 | .091 |
| 6 | Block | -.005 | .033 | 1451 | -0.155 | .877 |
| 6 | Exp.Response | -.072 | .047 | 151.9 | -1.534 | .127 |
| 6 | Adults | -.308 | .054 | 179.4 | -5.663 | <.001 |
| 6 | NoFP:Block | -.004 | .047 | 1458 | -0.083 | .934 |
| 6 | Pre-obj:Block | .071 | .046 | 1450 | 1.533 | .126 |
| 6 | NoFP:Exp.Response | .366 | .068 | 75.7 | 5.373 | <.001 |
| 6 | Pre-obj:Exp.Response | .230 | .067 | 151.9 | 3.446 | .001 |
| 6 | Block:Exp.Response | .049 | .046 | 1450 | 1.061 | .289 |
| 6 | NoFP:Adults | .173 | .065 | 1449 | 2.663 | .008 |
| 6 | Pre-obj:Adults | .081 | .064 | 1448 | 1.259 | .208 |
| 6 | Block:Adults | .051 | .046 | 1452 | 1.113 | .266 |
| 6 | Exp.Response:Adults | .031 | .064 | 1448 | 0.48 | .632 |
| 6 | NoFP:Block:Exp.Response | -.035 | .066 | 1455 | -0.534 | .594 |
| 6 | Pre-obj:Block:Exp.Response | -.090 | .065 | 1451 | -1.378 | .168 |
| 6 | NoFP:Block:Adults | -.115 | .065 | 1454 | -1.761 | .078 |
| 6 | Pre-obj:Block:Adults | -.129 | .064 | 1449 | -2.005 | .045 |
| 6 | NoFP:Exp.Response:Adults | -.221 | .091 | 1449 | -2.41 | .016 |
| 6 | Pre-obj:Exp.Response:Adults | -.218 | .091 | 1448 | -2.399 | .017 |
| 6 | Block:Exp.Response:Adults | -.150 | .064 | 1449 | -2.333 | .020 |
| 6 | NoFP:Block:Exp.Response:Adults | .253 | .091 | 1452 | 2.763 | .006 |
| 6 | Pre-obj:Block:Exp.Response:Adults | .206 | .091 | 1450 | 2.269 | .023 |
| 7 | Intercept | .693 | .044 | 105.9 | 15.728 | <.001 |
| 7 | NoFP | -.257 | .065 | 69.2 | -3.963 | <.001 |
| 7 | Pre-obj | -.101 | .057 | 95.3 | -1.774 | .079 |
| 7 | Block | .019 | .034 | 1375 | 0.567 | .571 |
| 7 | Exp.Response | -.098 | .057 | 94.5 | -1.724 | .088 |
| 7 | Adults | -.241 | .053 | 224.2 | -4.547 | <.001 |
| 7 | NoFP:Block | .067 | .053 | 1398 | 1.276 | .202 |
| 7 | Pre-obj:Block | .018 | .047 | 1374 | 0.375 | .708 |
| 7 | NoFP:Exp.Response | .349 | .089 | 62.7 | 3.921 | <.001 |
| 7 | Pre-obj:Exp.Response | .177 | .080 | 94.2 | 2.207 | .030 |
| 7 | Block:Exp.Response | .051 | .047 | 1373 | 1.076 | .282 |
| 7 | NoFP:Adults | .087 | .072 | 1371 | 1.211 | .226 |
| 7 | Pre-obj:Adults | .013 | .066 | 1374 | 0.199 | .842 |
| 7 | Block:Adults | .007 | .046 | 1376 | 0.144 | .885 |
| 7 | Exp.Response:Adults | .007 | .066 | 1372 | 0.114 | .909 |
| 7 | NoFP:Block:Exp.Response | -.125 | .071 | 1403 | -1.762 | .078 |
| 7 | Pre-obj:Block:Exp.Response | -.130 | .067 | 1375 | -1.938 | .053 |
| 7 | NoFP:Block:Adults | -.134 | .072 | 1373 | -1.862 | .063 |
| 7 | Pre-obj:Block:Adults | -.024 | .066 | 1373 | -0.367 | .713 |
| 7 | NoFP:Exp.Response:Adults | -.073 | .098 | 1373 | -0.748 | .455 |
| 7 | Pre-obj:Exp.Response:Adults | -.063 | .093 | 1373 | -0.676 | .499 |
| 7 | Block:Exp.Response:Adults | -.106 | .066 | 1372 | -1.617 | .106 |
| 7 | NoFP:Block:Exp.Response:Adults | .227 | .098 | 1373 | 2.323 | .020 |
| 7 | Pre-obj:Block:Exp.Response:Adults | .165 | .093 | 1374 | 1.775 | .076 |

The formula used, lmer(prop_alt_set ~ 1 + sentence.type * block * exp.response * age + (1 | id) + (1 | item), data = dat), included random variance components for participants and items. Models allowing for individual differences (i.e. random slopes) in the within-participants factors did not converge. AGE and SENTENCE TYPE were coded as treatment contrasts with children and Pre-subj sentences as base. BLOCK and EXPECTED RESPONSE were coded with a sum contrast.
