# Supplementary material for: Children’s Comprehension of Sentences with Focus Particles and the Role of Cognitive Control: An Eye Tracking Study with German-Learning 4-Year-Olds
Source: PLoS One. 2016 Mar 1;11(3):e0149870. doi: 10.1371/journal.pone.0149870 (PMC4773164; doi:10.1371/journal.pone.0149870)
Supplement: S2 Table — (DOCX) [file pone.0149870.s002.docx]

**S2 Table. Model output for the fixed effects in Study 2.**

| Window | Predictor | Estimate | SE | df | t-value | p-value |
| --- | --- | --- | --- | --- | --- | --- |
| 0 | Intercept | .679 | .033 | 49.8 | 20.529 | <2e-16 |
| 0 | NoFP | -.068 | .050 | 29.7 | -1.375 | .179 |
| 0 | Pre-obj | .031 | .044 | 40.5 | 0.716 | .478 |
| 0 | Block | -.013 | .020 | 1488 | -0.627 | .531 |
| 0 | Exp.Response | -.102 | .044 | 40.4 | -2.325 | .025 |
| 0 | NoFP:Block | .031 | .028 | 1486 | 1.1 | .271 |
| 0 | Pre-obj:Block | .014 | .028 | 1487 | 0.497 | .619 |
| 0 | NoFP:Exp.Response | .159 | .070 | 29.7 | 2.255 | .032 |
| 0 | Pre-obj:Exp.Response | .066 | .062 | 40.6 | 1.066 | .293 |
| 0 | Block:Exp.Response | .004 | .028 | 1489 | 0.143 | .886 |
| 0 | NoFP:Block:Exp.Response | .009 | .040 | 1487 | 0.221 | .825 |
| 0 | Pre-obj:Block:Exp.Response | .014 | .040 | 1489 | 0.358 | .721 |
| 1 | Intercept | .679 | .031 | 61.8 | 21.767 | <.001 |
| 1 | NoFP | -.273 | .044 | 30.4 | -6.242 | <.001 |
| 1 | Pre-obj | .001 | .040 | 48.7 | 0.036 | .971 |
| 1 | Block | .030 | .022 | 1495 | 1.339 | .181 |
| 1 | Exp.Response | -.257 | .040 | 48.6 | -6.381 | <.001 |
| 1 | NoFP:Block | .012 | .031 | 1494 | 0.401 | .689 |
| 1 | Pre-obj:Block | -.047 | .032 | 1495 | -1.502 | .133 |
| 1 | NoFP:Exp.Response | .288 | .062 | 30.6 | 4.639 | <.001 |
| 1 | Pre-obj:Exp.Response | .144 | .057 | 48.8 | 2.535 | .015 |
| 1 | Block:Exp.Response | -.017 | .031 | 1496 | -0.545 | .586 |
| 1 | NoFP:Block:Exp.Response | -.030 | .044 | 1494 | -0.676 | .499 |
| 1 | Pre-obj:Block:Exp.Response | .083 | .045 | 1495 | 1.854 | .064 |
| 2 | Intercept | .359 | .032 | 52.9 | 11.18 | <.001 |
| 2 | NoFP | -.123 | .044 | 24.4 | -2.784 | .010 |
| 2 | Pre-obj | .031 | .040 | 36.7 | 0.767 | .448 |
| 2 | Block | .021 | .020 | 1488 | 1.013 | .311 |
| 2 | Exp.Response | -.119 | .040 | 36.5 | -2.988 | .005 |
| 2 | NoFP:Block | -.018 | .029 | 1485 | -0.625 | .532 |
| 2 | Pre-obj:Block | -.007 | .029 | 1486 | -0.24 | .810 |
| 2 | NoFP:Exp.Response | .159 | .062 | 24.5 | 2.55 | .017 |
| 2 | Pre-obj:Exp.Response | .106 | .056 | 36.8 | 1.88 | .068 |
| 2 | Block:Exp.Response | -.038 | .029 | 1489 | -1.312 | .190 |
| 2 | NoFP:Block:Exp.Response | .006 | .041 | 1486 | 0.152 | .880 |
| 2 | Pre-obj:Block:Exp.Response | .044 | .041 | 1487 | 1.075 | .282 |
| 3 | Intercept | .469 | .035 | 41.9 | 13.267 | <.001 |
| 3 | NoFP | -.240 | .053 | 25.3 | -4.492 | <.001 |
| 3 | Pre-obj | -.233 | .047 | 34.6 | -4.953 | <.001 |
| 3 | Block | -.002 | .021 | 1484 | -0.105 | .916 |
| 3 | Exp.Response | -.003 | .047 | 34.4 | -0.059 | .953 |
| 3 | NoFP:Block | -.037 | .030 | 1482 | -1.245 | .213 |
| 3 | Pre-obj:Block | -.047 | .030 | 1482 | -1.539 | .124 |
| 3 | NoFP:Exp.Response | .249 | .076 | 25.4 | 3.292 | .003 |
| 3 | Pre-obj:Exp.Response | .260 | .067 | 34.6 | 3.903 | <.001 |
| 3 | Block:Exp.Response | -.024 | .030 | 1485 | -0.805 | .421 |
| 3 | NoFP:Block:Exp.Response | .048 | .042 | 1482 | 1.141 | .254 |
| 3 | Pre-obj:Block:Exp.Response | .043 | .043 | 1484 | 1.009 | .313 |
| 4 | Intercept | .545 | .030 | 59.6 | 18.114 | <.001 |
| 4 | NoFP | -.189 | .038 | 22 | -4.97 | <.001 |
| 4 | Pre-obj | -.256 | .037 | 44.8 | -6.897 | <.001 |
| 4 | Block | -.018 | .022 | 1698 | -0.794 | .427 |
| 4 | Exp.Response | -.011 | .037 | 44.1 | -0.296 | .768 |
| 4 | NoFP:Block | -.004 | .031 | 1692 | -0.115 | .908 |
| 4 | Pre-obj:Block | .023 | .032 | 1693 | 0.712 | .476 |
| 4 | NoFP:Exp.Response | .316 | .055 | 23.9 | 5.749 | <.001 |
| 4 | Pre-obj:Exp.Response | .279 | .053 | 45.4 | 5.287 | <.001 |
| 4 | Block:Exp.Response | .032 | .032 | 1697 | 0.999 | .318 |
| 4 | NoFP:Block:Exp.Response | -.001 | .045 | 1691 | -0.018 | .985 |
| 4 | Pre-obj:Block:Exp.Response | -.055 | .046 | 1692 | -1.204 | .229 |
| 5 | Intercept | .542 | .032 | 47 | 16.734 | <.001 |
| 5 | NoFP | -.175 | .047 | 24.2 | -3.762 | .001 |
| 5 | Pre-obj | -.162 | .042 | 35.7 | -3.864 | <.001 |
| 5 | Block | -.023 | .021 | 1985 | -1.095 | .274 |
| 5 | Exp.Response | -.077 | .042 | 35.4 | -1.845 | .073 |
| 5 | NoFP:Block | .019 | .029 | 1980 | 0.67 | .503 |
| 5 | Pre-obj:Block | .005 | .029 | 1981 | 0.162 | .871 |
| 5 | NoFP:Exp.Response | .363 | .066 | 24.3 | 5.514 | <.001 |
| 5 | Pre-obj:Exp.Response | .255 | .059 | 35.7 | 4.299 | <.001 |
| 5 | Block:Exp.Response | .047 | .029 | 1980 | 1.622 | .105 |
| 5 | NoFP:Block:Exp.Response | .005 | .041 | 1979 | 0.119 | .905 |
| 5 | Pre-obj:Block:Exp.Response | -.051 | .041 | 1982 | -1.223 | .221 |
| 6 | Intercept | .572 | .037 | 43.2 | 15.392 | <.001 |
| 6 | NoFP | -.237 | .057 | 26.8 | -4.181 | <.001 |
| 6 | Pre-obj | -.159 | .049 | 34 | -3.229 | .003 |
| 6 | Block | .017 | .022 | 1657.8 | 0.736 | .462 |
| 6 | Exp.Response | -.087 | .050 | 35.5 | -1.748 | .089 |
| 6 | NoFP:Block | -.015 | .032 | 1651 | -0.47 | .638 |
| 6 | Pre-obj:Block | -.029 | .031 | 1654.8 | -0.941 | .347 |
| 6 | NoFP:Exp.Response | .401 | .080 | 26.9 | 5.007 | <.001 |
| 6 | Pre-obj:Exp.Response | .225 | .070 | 34.6 | 3.22 | .003 |
| 6 | Block:Exp.Response | -.036 | .032 | 1656.7 | -1.146 | .252 |
| 6 | NoFP:Block:Exp.Response | .040 | .045 | 1649.1 | 0.883 | .377 |
| 6 | Pre-obj:Block:Exp.Response | .042 | .044 | 1651.5 | 0.954 | .340 |
| 7 | Intercept | .609 | .037 | 40.2 | 16.621 | <.001 |
| 7 | NoFP | -.192 | .054 | 23.1 | -3.541 | .002 |
| 7 | Pre-obj | -.155 | .047 | 29.9 | -3.277 | .003 |
| 7 | Block | -.009 | .026 | 1186 | -0.359 | .720 |
| 7 | Exp.Response | -.097 | .050 | 35.7 | -1.958 | .058 |
| 7 | NoFP:Block | .072 | .038 | 1180 | 1.898 | .058 |
| 7 | Pre-obj:Block | .013 | .035 | 1186 | 0.381 | .703 |
| 7 | NoFP:Exp.Response | .360 | .078 | 24.2 | 4.634 | <.001 |
| 7 | Pre-obj:Exp.Response | .223 | .069 | 33.5 | 3.227 | .003 |
| 7 | Block:Exp.Response | -.064 | .038 | 1189 | -1.674 | .094 |
| 7 | NoFP:Block:Exp.Response | .022 | .055 | 1182 | 0.407 | .684 |
| 7 | Pre-obj:Block:Exp.Response | .063 | .052 | 1188 | 1.193 | .233 |

The formula for the model specification is the same as for Study 1 (see S1 Table), only that the predictor Age is not included.
