# Supplementary material for: Children’s Comprehension of Sentences with Focus Particles and the Role of Cognitive Control: An Eye Tracking Study with German-Learning 4-Year-Olds
Source: PLoS One. 2016 Mar 1;11(3):e0149870. doi: 10.1371/journal.pone.0149870 (PMC4773164; doi:10.1371/journal.pone.0149870)
Supplement: S3 Table — (DOCX) [file pone.0149870.s003.docx]

**S3 Table. Model output for the fixed effects of the influence of the flanker task on the sentence-picture verfication in Study 2.**

| Effect | Estimate(logit) | Estimate (proportion) | SE | z-value | p-value |
| --- | --- | --- | --- | --- | --- |
| Intercept | 1.7648 | 0.6885097 | 0.4769 | 3.7 | .000215 |
| Pre-subj | -2.695 | -0.244808 | 0.3957 | -6.811 | <.0001 |
| Inteference effect centered | -0.9111 | -0.1453705 | 0.9923 | -0.918 | .358549 |
| Age in months centered | 0.2741 | 0.0188158 | 0.1814 | 1.511 | .13087 |
| Digit span centered | 3.0686 | 0.344751 | 0.741 | 4.141 | <.0001 |
| Pre-subj: Inteference effect | -3.2291 | -0.2093595 | 1.1547 | -2.797 | .005164 |
| Pre-subj:Age | -0.1897 | 0.0008694 | 0.1413 | -1.343 | .179335 |
| Pre-subj:Digit span | -0.1235 | -0.0632601 | 0.6278 | -0.197 | .844099 |

The formula used, glmer(accuracy ~ 1 + focus * (interference + ages.c + digitspan.c) + (1 |id) + (1 |item), data = dat, family=binomial(link = "logit")), included random variance components for participants and items. Focus position was coded as treatment contrast with Pre-obj as base. We centered the three predictors interference effect, age (in months), and digit span. The estimates as proportions are given in addition to the original logit estimates for ease of interpretation.
