# Supplementary material for: Children’s Comprehension of Sentences with Focus Particles and the Role of Cognitive Control: An Eye Tracking Study with German-Learning 4-Year-Olds
Source: PLoS One. 2016 Mar 1;11(3):e0149870. doi: 10.1371/journal.pone.0149870 (PMC4773164; doi:10.1371/journal.pone.0149870)
Supplement: S4 Table — (DOCX) [file pone.0149870.s004.docx]

**S4 Table. Complete sentence material used in Study 1 and Study 2.**

| Sentence type | Correct response | Sentence |
| --- | --- | --- |
| Pre-subj | yes | Nur der Elefant hat einen Drachen. |
| Pre-obj | yes | Der Elefant hat nur einen Drachen. |
| Pre-subj | yes | Nur die Ente hat einen Farbkasten. |
| Pre-obj | yes | Die Ente hat nur einen Farbkasten. |
| Pre-subj | yes | Nur die Ente hat einen Teddy. |
| Pre-obj | yes | Die Ente hat nur einen Teddy. |
| Pre-subj | yes | Nur der Elefant hat einen Schlitten. |
| Pre-obj | yes | Der Elefant hat nur einen Schlitten. |
| Pre-subj | yes | Nur der Maulwurf hat einen Roller. |
| Pre-obj | yes | Der Maulwurf hat nur einen Roller. |
| Pre-subj | yes | Nur der Maulwurf hat eine Schaufel. |
| Pre-obj | yes | Der Maulwurf hat nur eine Schaufel. |
| Pre-subj | yes | Nur die Maus hat einen Traktor. |
| Pre-obj | yes | Die Maus hat nur einen Traktor. |
| Pre-subj | yes | Nur die Maus hat eine Gitarre. |
| Pre-obj | yes | Die Maus hat nur eine Gitarre. |
| Pre-subj | no | Nur die Ente hat ein Boot. |
| Pre-obj | no | Die Ente hat nur ein Boot. |
| Pre-subj | no | Nur die Ente hat ein Buch. |
| Pre-obj | no | Die Ente hat nur ein Buch. |
| Pre-subj | no | Nur der Elefant hat eine Tasche. |
| Pre-obj | no | Der Elefant hat nur eine Tasche. |
| Pre-subj | no | Nur der Elefant hat eine Eisenbahn. |
| Pre-obj | no | Der Elefant hat nur eine Eisenbahn. |
| Pre-subj | no | Nur der Maulwurf hat einen Regenschirm. |
| Pre-obj | no | Der Maulwurf hat nur einen Regenschirm. |
| Pre-subj | no | Nur der Maulwurf hat einen Ball. |
| Pre-obj | no | Der Maulwurf hat nur einen Ball. |
| Pre-subj | no | Nur die Maus hat eine Laterne. |
| Pre-obj | no | Die Maus hat nur eine Laterne. |
| Pre-subj | no | Nur die Maus hat einen Hampelmann. |
| Pre-obj | no | Die Maus hat nur einen Hampelmann. |
| NoFP | no | Die Ente hat einen Würfel. |
| NoFP | yes | Die Ente hat einen Pinsel. |
| NoFP | no | Der Elefant hat einen Keks. |
| NoFP | yes | Der Elefant hat eine Banane. |
| NoFP | no | Der Maulwurf hat einen Topf. |
| NoFP | yes | Der Maulwurf hat eine Blume. |
| NoFP | yes | Die Maus hat eine Schere. |
| NoFP | no | Die Maus hat eine Mütze. |
